# Exploring the causal effects of serum lipids and lipidomes on lewy body dementia: a Mendelian randomization study

**DOI:** 10.3389/fendo.2024.1456005

**Published:** 2024-09-19

**Authors:** Qingan Fu, Guanrui Pan, Qingyun Yu, Zhekang Liu, Tianzhou Shen, Xiaowei Ma, Long Jiang

**Affiliations:** ^1^ Department of Cardiovascular Medicine, The Second Affiliated Hospital of Nanchang University, Nanchang, Jiangxi, China; ^2^ Rheumatology and Immunology Department, The Second Affiliated Hospital of Nanchang University, Nanchang, Jiangxi, China

**Keywords:** lewy body dementia, apolipoprotein E, serum lipid, lipidomes, Mendelian randomization, causal effect

## Abstract

**Background:**

Lewy body dementia (LBD) is a neurodegenerative disorder characterized by the accumulation of Lewy bodies, which primarily composed of misfolded alpha-synuclein (αS). The development of LBD and APOE4 subtypes is thought to be associated with disorders of lipid metabolism. In this study, we investigated the causal relationship between serum lipids, liposomes and LBD using a two-sample Mendelian randomization (TSMR) method.

**Methods:**

A TSMR analysis of genome-wide association study (GWAS) data for 8 serum lipids, 179 lipidomes components, LBD and its subtypes was performed, using inverse variance weighted as the primary outcome. To ensure robustness, the sensitivity analyses including MR Pleiotropy RESidual Sum and Outlier, Cochran’s test, leave-one-out method and funnel plots were performed.

**Results:**

In this study, we found that low-density lipoprotein cholesterol (LDL-C) (OR=1.45, 95% CI=1.19-1.77, P<0.001) and remnant cholesterol (RC) (OR=2.64, 95% CI=1.64-4.28, P<0.001) had significant positive causal effects on LBD, and RC also had a positive effect on LBD in carriers of the APOE4 gene. The results of lipidome analysis showed that phosphatidylcholine (PC) (O-16:0_20:4) levels (OR=0.86, 95% CI=0.75-0.98, P=0.02) and PC (O-18:1_20:4) levels (OR=0.76, 95% CI=0.65-0.89, P <0.001) had negative causal effects on LBD, whereas phosphatidylinositol (PI) (18:1_20:4) levels had a positive causal effect on LBD (OR=1.19, 95% CI=1.02-1.39, P=0.03). For LBD with APOE4 carriers, high levels of PC (16:1_18:0) and PC (O-18:2_18:1) had a significant positive effect, while high levels of PC (O-16:1_18:0), phosphatidylethanolamine (PE) (O-18:2_18:1), sphingomyelin (SM) (d38:2), and triacylglycerol (TAG) (56:5) significantly reduced the risk. No heterogeneity and horizontal pleiotropy were observed in sensitivity analysis.

**Conclusion:**

Elevated LDL-C and RC levels are significant risk factors for LBD, with RC also impacting APOE4-carrying LBD. Glycerophospholipids play a crucial role in the pathogenesis of LBD, but the specific components that play a role differ from those with the APOE4 carries. These findings highlight the importance of lipid metabolism in LBD and APOE4 subtypes.

## Introduction

Lewy body dementia (LBD) is a neurodegenerative disease, which is the second most common form of clinical dementia after Alzheimer’s disease, accounting for 4-8% of all dementia cases (1, 2). The formation of Lewy bodies, which are intracellular deposits that form in dopaminergic neurons of the central nervous system, is a unique pathological feature of LBD (3). The primary constituent of Lewy bodies is the misfolded, aggregated form of alpha-synuclein (αS), which is found in pathogenic inclusions (4). The main clinical features of LBD include early fluctuations in attention, hallucinations, and Parkinson’s syndrome (5). Existing studies have shown that LBD occurs in elderly individuals, with the majority of patients presenting with clinical symptoms between the ages of 70 and 85 years (6). The aging of the world population is increasing rapidly, the number of patients with LBD will continue to increase, creating a greater demand for care and a growing burden on health care resources globally (3). Therefore, an in-depth investigation of the pathogenesis of LBD and the identification of potential risk factors for LBD are imperative.

There is a special isoform of LBD, called the apolipoprotein E (APOE) allele-carrying LBD isoforms. The apolipoprotein E (APOE) gene, which is involved in lipid transport and metabolism, mainly has three different alleles (APOE2, APOE3, APOE4) in human being (7). In particular, carriers of the APOE4 allele are usually prone to lipid metabolism disorders are therefore more susceptible to other diseases (8–10). The most common diseases associated with APOE4 allele include Alzheimer’s disease and cardiovascular disease (11). Furthermore, recent studies have indicated that the APOE4 allele status in Parkinson’s disease (PD) may be an important predictor of cognitive decline in Parkinson’s disease, its effect appears to be independent of gender, as in the findings of Umeh et al. (12). In addition, increased aggregation of αS proteins in the brains of LBD patients is strongly associated with carrying the APOE4 allele (13). However, to date, no study has clearly demonstrated the potential relationship between lipids and disease risk in LBD patients carrying the APOE4 allele or whether this potential relationship is associated with abnormal changes in the αS protein.

Recently, serum lipid levels have been shown to be associated with the occurrence and development of LBD. In a previous cross-sectional study, it was found that higher serum low-density lipoprotein cholesterol (LDL-C) concentration and lower high-density lipoprotein cholesterol (HDL-C) concentration will lead to an increased risk of LBD (14). Another Mendelian randomization (MR) study also confirmed the positive genetic causal effect of serum LDL-C level on the LBD risk (15). However, the existing studies included fewer types of lipids and did not comprehensively explore lipids composition. Lipidome components such as phospholipids and cholesterol are major sources of cell membrane components. Changes in cell membrane components have been shown to promote the aggregation of αS into amyloidogenic fibrils (16). Studies demonstrates that specific lipid fractions may also have an important part to play in the pathogenesis of LBD (17). However, few research studies have investigated the link between lipid fractions and LBD.

MR is predominantly used to explore causal relationships between exposures phenotypes and outcomes phenotypes from a genetic perspective. It can minimize the impact of confounders and the interference of negative causal effects (18, 19). Preliminary research has been conducted in previous studies to examine the effect of common lipids on the Lewy bodies dementia, but these studies remain incomplete. Therefore, the present study used a two-sample MR (TSMR) approach and try to provide insight into the causal effects of eight lipids and liposomes subdivided into 179 subfractions on LBD and its APOE4 gene-carrying subtypes. Hope the results could be instrumental for risk prediction, early prevention, and precision targeted therapy for LBD and its subtypes.

## Method

### Study design

**Figure 1:**
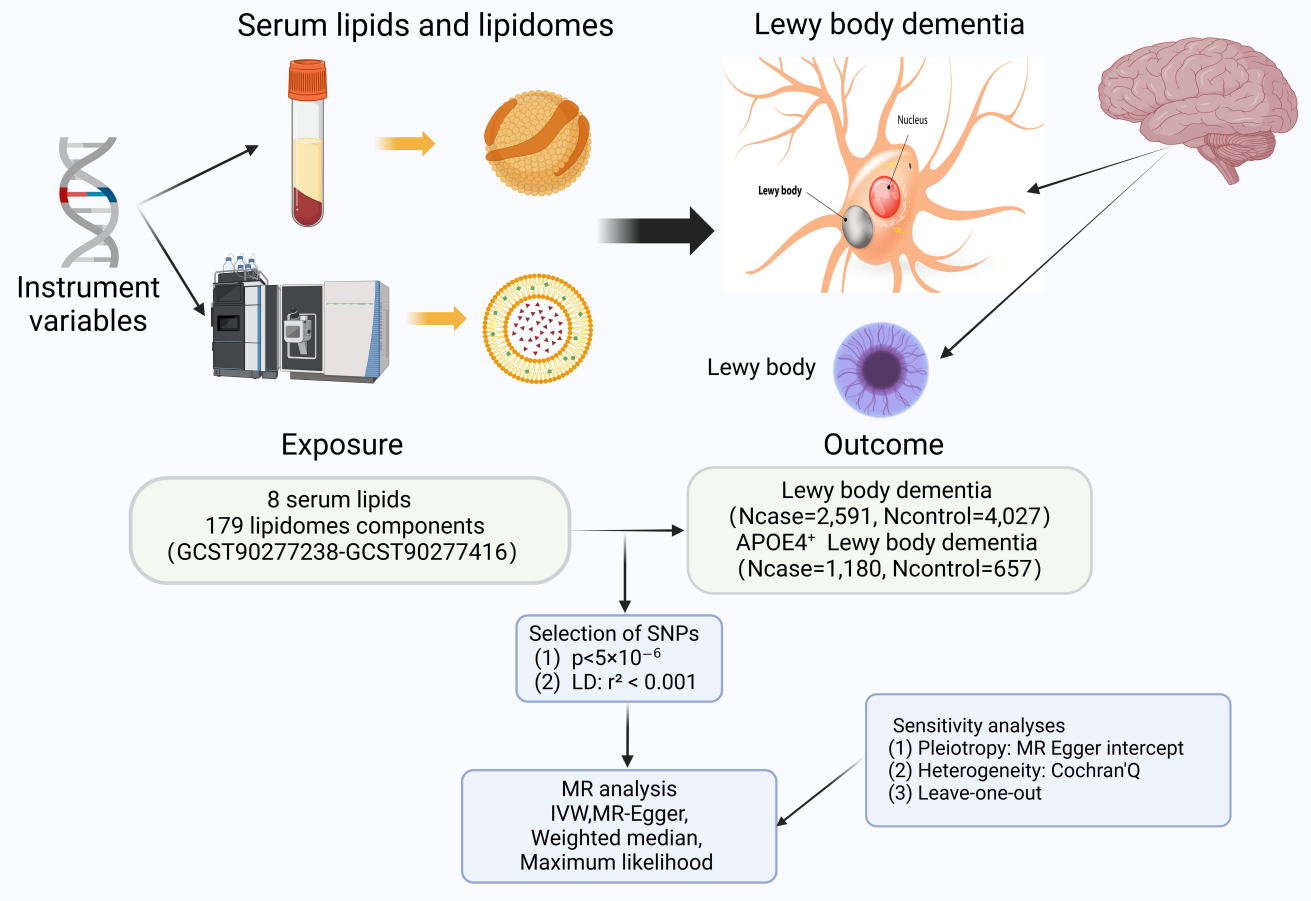
The flowchart contains a brief summary of the exposure, outcome, methodology, and sensitivity analyses for this two-sample Mendelian randomization; with some elements cited from https://alzheimersnewstoday.com. (Created with BioRender.com). APOE, apolipoprotein E; LD, linkage disequilibrium; MR, Mendelian randomization; SNP, Single-Nucleotide Polymorphism; IVW, Inverse variance weighted.

Among the existing studies, we explored causal associations between eight conventional lipids (LDL-C, TC, HDL-C, Lp(a), TG, APOB, RC and APOA) and a subdivided set of 179 lipidomes composition fractions with LBD or APOE4 gene-carrying subtypes of LBD based on a TSMR approach. Our study strictly followed three basic principles: (1) IVs are strongly associated with the exposure; (2) IVs are independent of confounder; (3) IVs are not associated with outcomes directly, only influence outcomes through exposures. A simple flowchart is presented in **Figure 1**, and the study design complied with the requirements of the Mendelian Randomization of Observational Studies with Enhanced Epidemiology Reporting (STROBE-MR) as described in the **Supplementary Materials** (20).

### Data sources

#### Sources of LBD GWAS data

GWAS data on LBD from a multicenter study (6), the researchers recruited 6,618 participants of European ancestry from multiple centers and cohorts in Europe and North America (Ncase=2,591, Ncontrol=4,027). In contrast, GWAS data for APOE 4 gene-carrying-positive LBD came from another study of LBD subtypes, which enrolled 1,180 patients and 657 healthy controls with a total of 5,912,161 SNPs (21).

#### Sources of serum lipids GWAS data

The GWAS data for the eight lipids analyzed in this paper were all retrieved in IEU OpenGWAS project: LDL-C (n=201,678), TC (n=344,278), HDL-C (n=403,943), TG (n=441,016), APOB (n=439,214), RC (n=115,078) and APOA (n=393,193). The majority of these GWAS data were from participants of European origin, all raw data studies were ethically reviewed and all participants signed informed consent forms, all GWAS dataset can be found in **Supplementary Table 1**.

#### Sources of 179 lipidomes GWAS data

The lipidomes dataset were obtained from a comprehensive GWAS study which performed mass spectrometry on 7174 Finnish participants (2595 males and 4579 females) based on the GeneRISK cohort (22). A total of 179 lipidomes were obtained in the study, which have been indexed in the GWAS database (registry numbers GCST90277238-GCST90277416). The data contained four major lipids: Glycerolipids (GL), Sphingolipids (SL), Glycerophospholipids (GP), and Sterols (ST), with a total of 13 lipid subclasses covered by the four lipids: GL: Triacylglycerol (TAG) n=38; Diacylglycerol (DAG) n=6; SL: Ceramide (Cer) n=4; Sphingomyelin (SM) n=11; GP: Phosphatidylinositol (PI) n=10; Phosphatidylethanolamine-ether (PEO) n=8; Phosphatidylethanolamine (PE) n=5; Phosphatidylcholine-ether (PCO) n=27; Phosphatidylcholine (PC) n=46; Lysophosphatidylethanolamine (LPE) n=3; Lysophosphatidylcholine (LPC) n=5; ST: Cholesteryl ester (CE) n=15; Cholesterol (Chol) n=1.

### Selection of instruments

The strict inclusion exclusion criteria was implemented when screening IVs, including only SNPs strongly associated with the exposure phenotype (P<5×10^-8^), excluding SNPs associated with the outcome (P<5×10^-6^), and further filtering IVs by chain imbalance (window size = 10,000 kb, r2 threshold = 0.001). We also removed duplicates, SNPs with missing information, and palindromic sequences and applied the PhenoScanner website to assess whether IVs were associated with other risk factors to avoid confounding effects. Evaluating the strength of effect of IVs and reducing bias, we calculated the F-value of IVs using the formula (F= R2*(N-2)/1-R), and excluded all weak IVs with an F<10 (23).

### Statistical analyses

In this study, Inverse Variance Weighted (IVW), Weighted Median, Maximum likelihood and MR-Egger were used to infer causality, the IVW results were the main results of TSMR (24). To avoid the results being affected by heterogeneity and horizontal pleiotropy, A series of sensitivity analyses were performed, and further removed outliers from the eligible SNPs using MR-PRESSO to avoid horizontal pleiotropy (25). The Q-value of the Cochrane test was used to detect heterogeneity in IV, and the symmetry of the funnel plot can indicate that horizontal pleiotropy is not significant (26). The MR-Egger’s intercept was used to test the heterogeneity of SNPs, and the same sensitivity analysis was performed in the reverse MR analysis to guarantee the reliability of the results. All the data in this study were analyzed through the “TwoSampleMR”, and “MR-PRESSO” packages (R version 4.3.0).

## Results

### Causal relationship between serum lipids and LBD and its APOE4 gene carrying subtype

**Figure 2:**
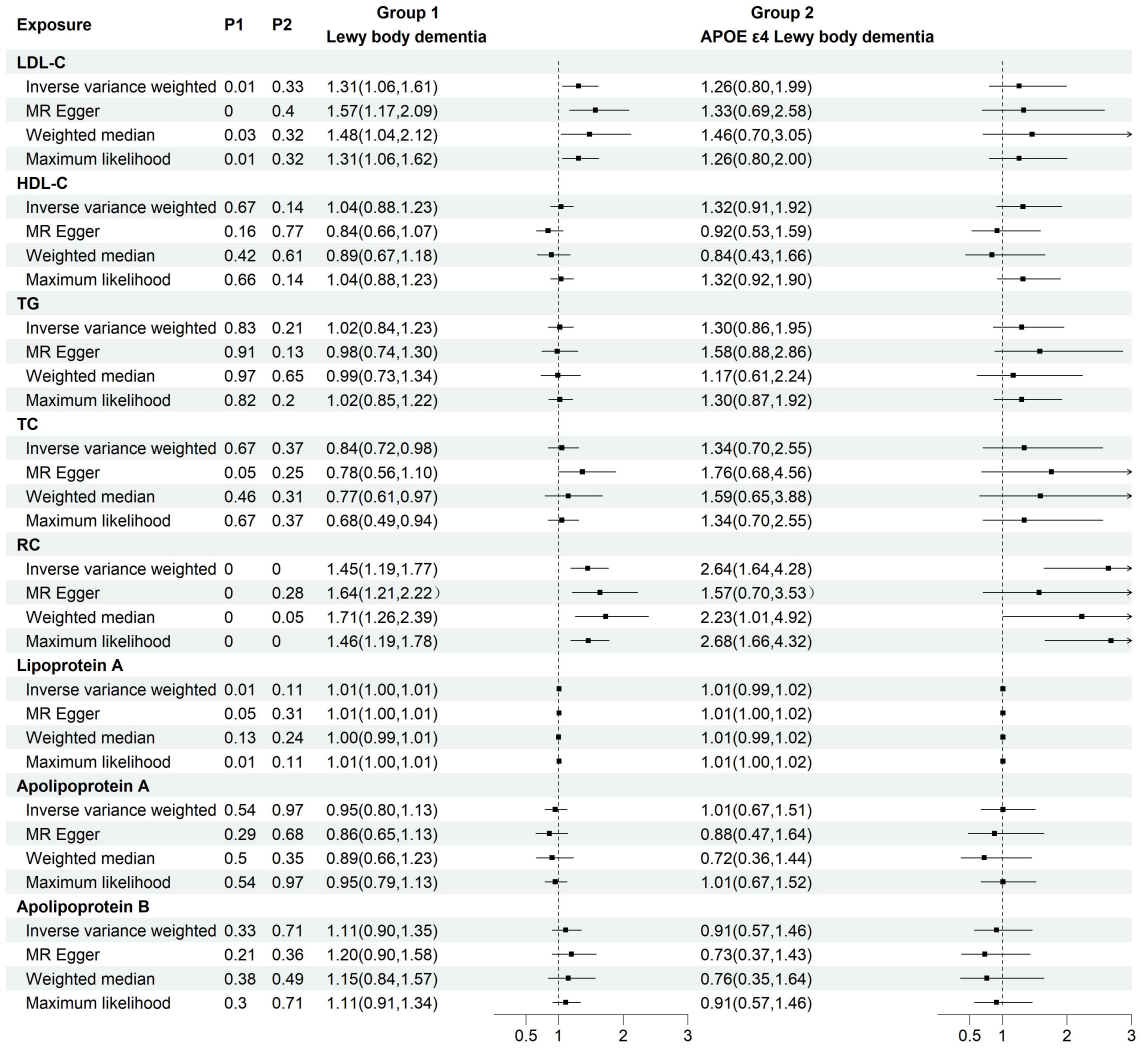
Two-group forest plot of TSMR results of the causal effect of eight serum lipids on LBD and APOE4 gene-carrying LBD. HDL-C, high-density lipoprotein cholesterol; LDL-C, low-density lipoprotein cholesterol; Lp(a), Lipoprotein (a); TC, total cholesterol; TG, triglyceride; RC, residual cholesterol; MR, Mendelian randomization.

To investigate the causal relationship between serum lipids and LBD and APOE4 gene-carrying subtypes, we first performed TSMR analyses with eight lipid components as exposures and LBD as well as APOE4 gene-carrying LBD subtypes as outcomes. The results of the analysis showed a significant positive causal effect of LDL-C (OR=1.45, 95% CI=1.19-1.77, P<0.001) and RC (OR=1.45, 95%CI=1.19-1.77, P<0.001) on the development of LBD, whereas only RC had a positive causal effect on the development of APOE4-carrying LBD (OR=2.64, 95%CI=1.64-4.28, P<0.001). (**Figure 2**). To avoid the influence of reverse causality effect, reverse TSMR analysis was then performed with LBD and APOE4 gene harboring LBD as exposure and lipids as outcome, and did not find any significant effect of LBD and subtypes on the any lipid component (**Supplementary Table 2**). Subsequently, a series of sensitivity analyses were performed. The results showed Cochran’s Q test did not find explicit heterogeneity, while the leave-one-out method and funnel plot demonstrated that the results were not affected by single SNPs and horizontal pleiotropy (**Supplementary Table 3**, **Supplementary Figures S1**-**S3**).

### Causal effects of lipidomes on LBD risk

**Figure 3:**
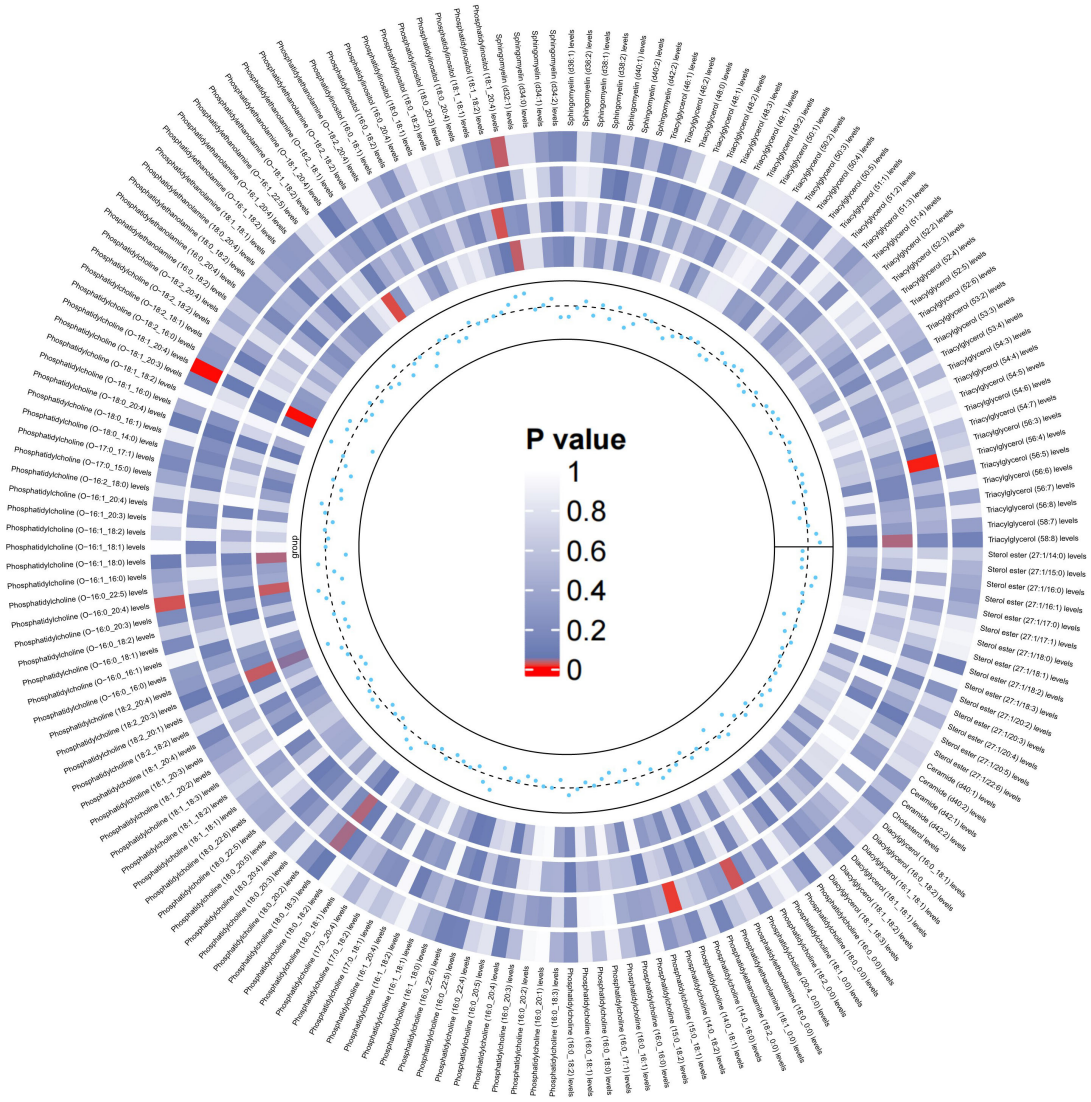
Circular heatmap of the causal effect of 179 liposomes components on LBD, with color shades representing the magnitude of significance. The circular heat map represents the four MR methods, including IVW, MR-Egger, Weighted Median, Maximum likelihood, in order from the outer ring to the inner ring, and the innermost scatter distribution represents the direction of the causal effect.

On the basis of the results of eight lipid composition analyses, we recognized that the LDL-C and RC has a causal effect on LBD, but which lipid component plays a key role is still unclear. Therefore, the present study used data from 179 lipidomes to explore in depth the causal effect between lipid composition and LBD. As shown in **Figure 3**, when 179 lipidomes were used as the exposure, the results indicated that PC (O-16:0_20:4) levels (OR=0.86, 95% CI=0.75-0.98, P=0.02), PC (O-18:1_20:4) levels (OR=0.76, 95% CI=0.65-0.89, P <0.001) had a negative causal effect on LBD, whereas PI (18:1_20:4) levels (OR=1.19, 95% CI=1.02-1.39, P=0.03) had a positive causal effect on LBD (**Supplementary Table 4**). Subsequent sensitivity analyses of the TSMR results in Cochran’s Q test for PC (O-16:0_20:4) (Q=23.91, P=0.47), PC (O-18:1_20:4) (Q=20.26,P=0.44) and PI (18:1_20:4) (Q=23.21,P=0.51), indicated that there is no significant heterogeneity in our results, and subsequent sensitivity analyses proved that there is no horizontal pleiotropy (**Supplementary Table 5**, **Supplementary Figures S4**-**S6**).

### Causal effects of lipidomes on APOE4 gene carrying LBD risk

**Figure 4:**
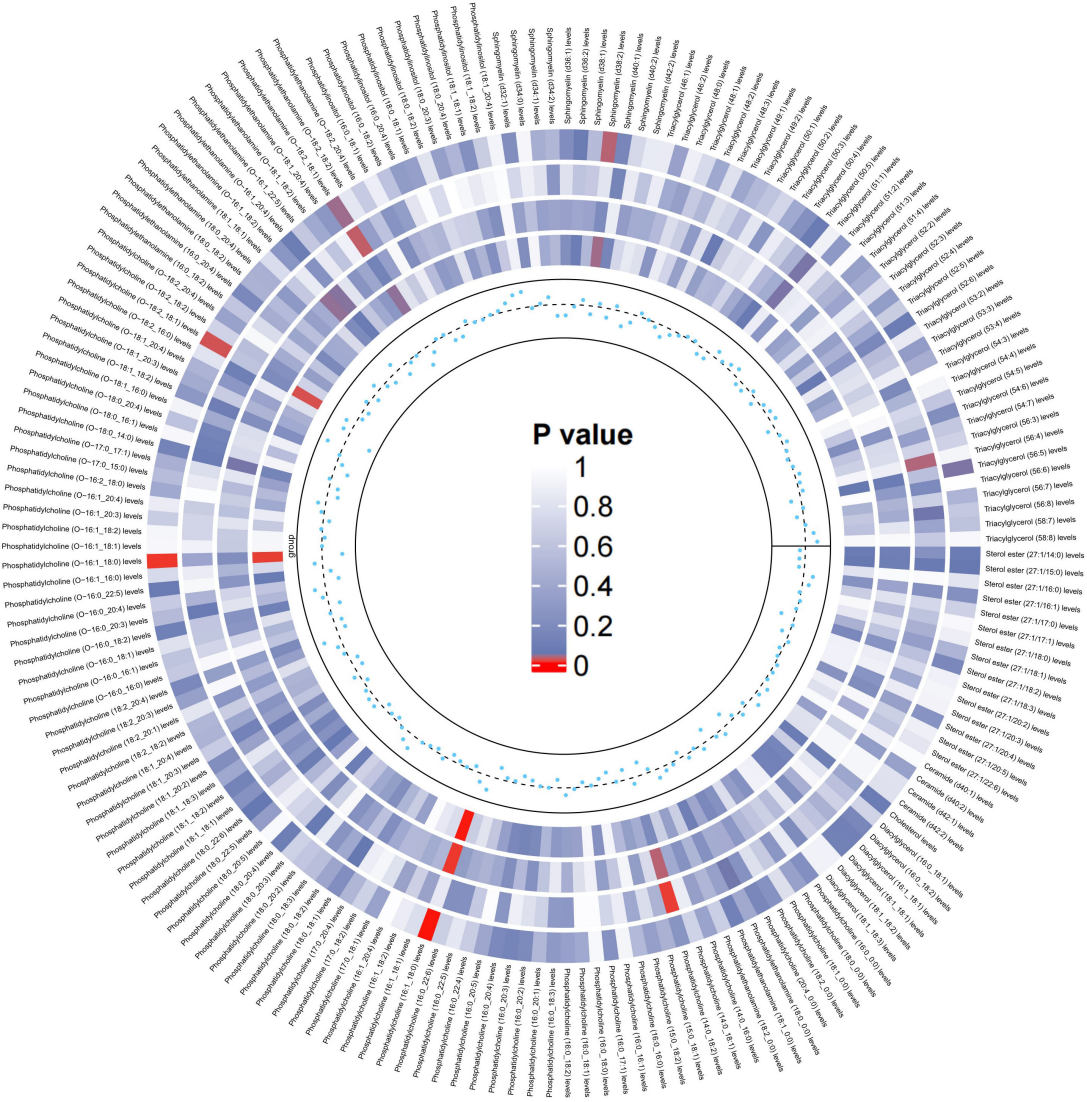
Circular heatmap of the causal effect of 179 lipidomes components on the LBD carried by the APOE4 gene, with color shades representing the magnitude of significance. The circular heat map represents the results of four MR methods, including IVW, MR-Egger, Weighted Median, Maximum likelihood, in order from the outer ring to the inner ring, and the innermost scatter distribution represents the direction of the causal effect.

Similarly, we used the lipidomes as an exposure and APOE4 gene-carrying LBD as an outcome to explore the causal effect of the lipidomes on the latter, and the results are shown in **Figure 4**. The MR analysis results are dominated by the IVW method, which in the figure is located in the outermost circle of the image, with brighter red color representing a more statistically significant IVW result. It can be observed that the lipid components related to APOE4 gene-carrying LBD is different to LBD, with higher PC (16:1_18:0) (OR=2.05, 95% CI=1.31-3.19, P=0.001) and PC (O-18:2_18:1) (OR=1.62, 95% CI=1.08-2.45, P=0.02) as significant risk factors for the development of APOE4 gene-carrying LBD. Meanwhile higher PC (O-16:1_18:0) (OR=0.50, 95% CI=0.30-0.84 P=0.01), PE (O-18:2_18:1) (OR=0.70, 95% CI=0.49-0.98, P=0.04), SM (d38:2) (OR=0.68, 95% CI=0.48-0.97, P=0.03) and TAG (56:5) levels (OR=0.70, 95% CI=0.50-0.99, P=0.04), on the other hand, were able to significantly reduce the risk of developing APOE4 gene-carrying LBD (**Supplementary Table 6**). The sensitivity analyses showed no significant heterogeneity was observed in the MR-Egger and Cochran’s Q test for IVW, and the leave-one-out results and funnel plots with symmetric distribution demonstrated the absence of aberrant SNPs and horizontal pleiotropy (**Supplementary Table 7**, **Supplementary Figures S7–S12**).

## Discussion

This is the first study to systematically assess the causal relationship between *in vivo* lipids, lipid composition and the risk of LBD and LBD with APOE4 alleles. First, our findings suggest that higher LDL-C and RC levels significant cause an increased risk of LBD. Second, among the LBD subtypes of APOE4 gene carriers, only RC levels have a causal positive relationship. Third, elevated levels of PC (18:1_20:4) increase the risk of LBD, while elevated levels of PC (O-16:0 20:4) and PC (O-18:1_20:4) have protective effect for LBD. Fourth, for LBD patients with APOE4 alleles, elevated levels of PC (16:1 18:0) and PC (O-18:2_18:1) lead to an increased risk, and elevated levels of PC (O-16:1_18:0), PEO (O-18:2_18:1), SM (d38:2), and TAG (56:5) significantly reduce the risk. Our study reveals the causal effects of multiple lipids on LBD and its APOE4 subtypes, enriching researchers’ understanding of lipids in the pathogenesis of LBD disease.

The results of the present study are consistent with the findings of previous studies showing that elevated LDL-C and RC levels are positively and causally associated with the development of LBD (15). Previous studies have shown that the vast majority of cholesterol in the brain is normally produced by astrocytes and oligodendrocytes. The blood-brain barrier (BBB) prevents potentially neurotoxic peripheral serum cholesterol from entering the brain, thus protecting neuronal function (27). In patients with hypercholesterolemia, researchers have observed an increase in serum–brain barrier permeability with increasing serum cholesterol concentrations, including LDL-C and RC, to cross the serum–brain barrier and enter and accumulate in the central nervous system (28, 29). Since the brain is unable to degrade cholesterol, this excess cholesterol is mainly excreted by oxidizing cholesterol to produce oxysterols. Among these, 27-hydroxycholesterol (27-OHC), a class of oxysterols, plays an important role in promoting the aggregation and diffusion of αS (30, 31). The abnormal aggregation of αS is usually considered one of the main pathological features of LBD. In contrast, we did not observe any causal relationship between LDL-C and APOE4 allele-carrying LBD, and elevated RC levels were the only risk factor for APOE4-carrying LBD. This finding suggested that LDL-C is not a risk factor for neurodegenerative diseases in individuals with APOE4 carriage but is only an independent risk factor for LBD risk. This conclusion is supported by a recent study that concluded that elevated RC levels have a potentially stronger role in APOE4-associated dementia risk than do common lipid components (e.g., TC and LDL-C). In addition, another study showed that LDL-C is similarly unrelated to APOE genotypes in the pathophysiology of Alzheimer’s disease (32). However, the exact mechanisms need to be further investigated (33).

The interpretation of the causal effect of lipid composition on LBD and the APOE4 gene is more complex and involves mainly the interaction of αS with the lipid components of phospholipid membranes (34). When αS binds and interacts with lipid membranes, αS undergoes a conformational change, i.e., the formation of insoluble oligomers by increasing the α-helix content, which in turn leads to the development of LBD (35). Many studies have shown that the relationship between the action of αS and lipid membranes depends on the lipid composition of the membrane, and our findings provide strong support for this view. PC, one of the most abundant phospholipids in cell membranes, has the complex effect on LBD and APOE4 allele-carrying LBD in this study. The presence of PC resulted in a decreased parallel β-folds in the secondary structures of oligomers, while the number of α-helices and disordered protein secondary structures increased. The interaction between αS and PC may also alter the structure and function of cell membranes (36). And in a lipidomic study of Parkinson’s disease, which cerebrospinal fluid from patients with PD was shown to contain increased levels of PCs, including PC (O-18:3_20:3), PC (14:0_18:2) and PC (O-20:2_24:3) (37). Our findings also revealed that in the case of APOE4 allele carriage, the presence of other phospholipids, including PIs and SMs may also be closely associated with the pathogenesis of disease. SMs are implicated in the pathogenesis of αS, leading to increased αS expression and affecting its membrane binding and aggregation in neurons (38). The presence of PI in phospholipid vesicles significantly increases the binding of soluble αS to the membrane and leads to extensive phospholipid bilayer disruption and aggregate formation (39). However, the specific mechanism needs further study.

Our findings support that LDL-C and RC are high risk factors for LBD, and dietary modification of LDL-C and RC levels in addition to medications may have a positive impact on prognostic outcomes. In a recent study, APOE4 carriers with higher dietary cholesterol intake were found to have a poorer lipid profile, which was associated with a higher risk of dementia and cognitive impairment. These associations were not observed in non-APOE4 allele carriers. The findings suggest that unfavorable lipid profile may be an important clinical indicator of dementia risk, especially in individuals with the APOE4 genotype. Dietary modifications to reduce the risk of dementia in the early stages of the disease include reducing the intake of saturated fats, trans fats and cholesterol to achieve healthy lipid levels (32). However, the current evidence does not sufficiently validate the use of omega-3 fatty acid supplements as a treatment for Alzheimer’s disease (40). Moreover, their efficacy in diminishing the occurrence of Alzheimer’s is also not convincingly demonstrated (41). In addition, the composition and structure of cell membrane lipid components can be altered in a targeted therapeutic manner, such as by up- or downregulating the expression of specific lipids, enzymes, or transcription factors, to treat disease, an approach known as membrane lipid therapy (MLT). For example, docosahexaenoic acid (DHA) has been used in research to treat Alzheimer’s disease. By design, 2-hydroxy-DHA (LP226A1, Lipopharma) was tested in a severe Alzheimer’s disease model animals (5XFAD mice). And 4 months of treatment with this synthetic unsaturated fatty acid increased new neuron production and restored cognitive scores on the radial maze test to control values (42). Our study identified lipidome components that are closely associated with the development of LBD and APOE4 allele-carrying LBD. It will help researchers develop targeted precision therapies for LBD in the future.

The advantage of this study is that the LBD GWAS data used is the largest and only sample data set. Although the original data was from multicenter and the participants spanned Europe and the United States, no heterogeneity, pleiotropy, and reverse causality were observed in a series of sensitivity analyses and reverse MR, which ensures the reliability of the results in our research. Similarly, the shortcomings of the study should not be overlooked. First, it only established causality between certain lipids and LBD or the APOE4 allele, without delving into mechanisms which need further basic research. Second, due to the limitations of GWAS data, we were unable to perform detailed subgroup analyses based on sex and age, and new GWAS data containing variables such as sex and age need to be utilized for more personalized subgroup studies in the future. Finally, the GWAS data for the lipidomes used in the study were from the Finnish people. Therefore, it needs to be further verified by large multicenter RCT studies in different ethnicities and regions.

## Conclusion

In this TSMR study, our findings provide evidence for a causal relationship between lipids, lipid composition and the risk of LBD and APOE allele-carrying LBD. The results suggest that certain lipids, such as LDL-C, RC, PI, and some PCs, are associated with increased LBD risk, while some subset of PCs may offer protection. In addition, different lipid components also affect the risk of APOE allele-carrying LBD, but their components are completely different with LBD. Our findings pave the way for a better understanding of serum lipids’ impact on LBD and APOE allele-carrying LBD, hope to guide the development of specific treatments. However, further research is needed to clarify the detailed mechanisms of lipids influence on LBD and APOE allele-carrying LBD risk.

## Data availability statement

All GWAS data are publicly available, the original study has been reviewed by an ethics committee, all participants have signed an informed consent form, and further information about the data can be obtained by contacting the corresponding author.

## References

[1] Taylor JP McKeith IG Burn DJ Boeve BF Weintraub D Bamford C. New evidence on the management of Lewy body dementia. Lancet Neurol. (2020) 19:157–69. doi: 10.1016/S1474-4422(19)30153-X PMC7017451 31519472

[2] Vann Jones SA O'Brien JT. The prevalence and incidence of dementia with Lewy bodies: a systematic review of population and clinical studies. Psychol Med. (2014) 44:673–83. doi: 10.1017/S0033291713000494 23521899

[3] Walker Z Possin KL Boeve BF Aarsland D. Lewy body dementias. Lancet. (2015) 386:1683–97. doi: 10.1016/S0140-6736(15)00462-6 PMC5792067 26595642

[4] Outeiro TF Koss DJ Erskine D Walker L Kurzawa-Akanbi M Burn D. Dementia with Lewy bodies: an update and outlook. Mol Neurodegener. (2019) 14:5. doi: 10.1186/s13024-019-0306-8 30665447 PMC6341685

[5] Bayram E Coughlin DG Rajmohan R Litvan I. Sex differences for clinical correlates of substantia nigra neuron loss in people with Lewy body pathology. Biol Sex Differ. (2024) 15:8. doi: 10.1186/s13293-024-00583-6 38243325 PMC10797801

[6] Chia R Sabir MS Bandres-Ciga S Saez-Atienzar S Reynolds RH Gustavsson E. Genome sequencing analysis identifies new loci associated with Lewy body dementia and provides insights into its genetic architecture. Nat Genet. (2021) 53:294–303. doi: 10.1038/s41588-021-00785-3 33589841 PMC7946812

[7] Yamazaki Y Zhao N Caulfield TR Liu CC Bu G. Apolipoprotein E and Alzheimer disease: pathobiology and targeting strategies. Nat Rev Neurol. (2019) 15:501–18. doi: 10.1038/s41582-019-0228-7 PMC7055192 31367008

[8] Tai LM Thomas R Marottoli FM Koster KP Kanekiyo T Morris AW. The role of APOE in cerebrovascular dysfunction. Acta Neuropathol. (2016) 131:709–23. doi: 10.1007/s00401-016-1547-z PMC4837016 26884068

[9] Masterman T Zhang Z Hellgren D Salter H Anvret M Lilius L. APOE genotypes and disease severity in multiple sclerosis. Mult Scler. (2002) 8:98–103. doi: 10.1191/1352458502ms787oa 11990879

[10] Koutsodendris N Nelson MR Rao A Huang Y. Apolipoprotein E and Alzheimer's disease: findings, hypotheses, and potential mechanisms. Annu Rev Pathol. (2022) 17:73–99. doi: 10.1146/annurev-pathmechdis-030421-112756 34460318

[11] Mahley RW. Apolipoprotein E: from cardiovascular disease to neurodegenerative disorders. J Mol Med (Berl). (2016) 94:739–46. doi: 10.1007/s00109-016-1427-y PMC4921111 27277824

[12] Umeh CC Mahajan A Mihailovic A Pontone GM. APOE4 allele, sex, and dementia risk in Parkinson's disease: lessons from a longitudinal cohort. J Geriatr Psychiatry Neurol. (2022) 35:810–5. doi: 10.1177/08919887211060019 PMC11062588 34958617

[13] Zhao N Attrebi ON Ren Y Qiao W Sonustun B Martens YA. APOE4 exacerbates α-synuclein pathology and related toxicity independent of amyloid. Sci Transl Med. (2020) 12:eaay1809. doi: 10.1126/scitranslmed.aay1809 PMC8309690 32024798

[14] Dou Y Liu S Li Y Wu H Chen H Ji Y. Plasma cholesterol levels as potential nutritional biomarkers for Lewy body dementia. J Alzheimers Dis. (2022) 86:779–86. doi: 10.3233/JAD-215295 35124646

[15] Liu P Liu J Zhang Y Xing X Zhou L Qu J. Elevated serum LDL-C increases the risk of Lewy body dementia: a two-sample mendelian randomization study. Lipids Health Dis. (2024) 23:42. doi: 10.1186/s12944-024-02032-0 38331880 PMC10851540

[16] Fusco G Chen SW Williamson PTF Cascella R Perni M Jarvis JA. Structural basis of membrane disruption and cellular toxicity by α-synuclein oligomers. Science. (2017) 358:1440–3. doi: 10.1126/science.aan6160 29242346

[17] Hallett PJ Engelender S Isacson O. Lipid and immune abnormalities causing age-dependent neurodegeneration and Parkinson's disease. J Neuroinflamm. (2019) 16:153. doi: 10.1186/s12974-019-1532-2 PMC6647317 31331333

[18] Fu Q Shen T Yu Q Jiang L Yang R. Causal effect of gallstone disease on the risk of coronary heart disease or acute myocardial infarction: a Mendelian randomization study. Sci Rep. (2023) 13:18807. doi: 10.1038/s41598-023-46117-9 37914780 PMC10620410

[19] Liu Z Shao Y Duan X. Genetic link between primary biliary cholangitis and connective tissue diseases in European populations: A two-sample Mendelian randomization study. PLoS One. (2024) 19:e0298225. doi: 10.1371/journal.pone.0298225 38335208 PMC10857725

[20] Davey Smith G DN Dimou N Egger M Gallo V Golub R Higgins JP. STROBE-MR: Guidelines for strengthening the reporting of Mendelian randomization studies. PeerJ Preprints. (2019) 7:e27857v1. doi: 10.7287/peerj.preprints.27857

[21] Kaivola K Shah Z Chia R Scholz SW. Genetic evaluation of dementia with Lewy bodies implicates distinct disease subgroups. Brain. (2022) 145:1757–62. doi: 10.1093/brain/awab402 PMC9423712 35381062

[22] Ottensmann L Tabassum R Ruotsalainen SE Gerl MJ Klose C Widén E. Genome-wide association analysis of plasma lipidome identifies 495 genetic associations. Nat Commun. (2023) 14:6934. doi: 10.1038/s41467-023-42532-8 37907536 PMC10618167

[23] Hemani G Bowden J Davey Smith G. Evaluating the potential role of pleiotropy in Mendelian randomization studies. Hum Mol Genet. (2018) 27:R195–r208. doi: 10.1093/hmg/ddy163 29771313 PMC6061876

[24] Burgess S Thompson SG. Interpreting findings from Mendelian randomization using the MR-Egger method. Eur J Epidemiol. (2017) 32:377–89. doi: 10.1007/s10654-017-0255-x PMC5506233 28527048

[25] Hemani G Zheng J Elsworth B Wade KH Haberland V Baird D. The MR-Base platform supports systematic causal inference across the human phenome. Elife. (2018) 7:e34408. doi: 10.7554/eLife.34408 PMC5976434 29846171

[26] Burgess S Zuber V Valdes-Marquez E Sun BB Hopewell JC. Mendelian randomization with fine-mapped genetic data: Choosing from large numbers of correlated instrumental variables. Genet Epidemiol. (2017) 41:714–25. doi: 10.1002/gepi.22077 PMC5725678 28944551

[27] Pfrieger FW. Cholesterol homeostasis and function in neurons of the central nervous system. Cell Mol Life Sci. (2003) 60:1158–71. doi: 10.1007/s00018-003-3018-7 PMC11138592 12861382

[28] Chan RB Oliveira TG Cortes EP Honig LS Duff KE Small SA. Comparative lipidomic analysis of mouse and human brain with Alzheimer disease. J Biol Chem. (2012) 287:2678–88. doi: 10.1074/jbc.M111.274142 PMC3268426 22134919

[29] Xue-Shan Z Juan P Qi W Zhong R Li-Hong P Zhi-Han T. Imbalanced cholesterol metabolism in Alzheimer's disease. Clin Chim Acta. (2016) 456:107–14. doi: 10.1016/j.cca.2016.02.024 26944571

[30] Doria M Maugest L Moreau T Lizard G Vejux A. Contribution of cholesterol and oxysterols to the pathophysiology of Parkinson's disease. Free Radic Biol Med. (2016) 101:393–400. doi: 10.1016/j.freeradbiomed.2016.10.008 27836779

[31] Dai L Wang J Zhang X Yan M Zhou L Zhang G. 27-hydroxycholesterol drives the spread of α-synuclein pathology in Parkinson's disease. Mov Disord. (2023) 38:2005–18. doi: 10.1002/mds.29577 37593929

[32] Wingo AP Vattathil SM Liu J Fan W Cutler DJ Levey AI. LDL cholesterol is associated with higher AD neuropathology burden independent of APOE. J Neurol Neurosurg Psychiatry. (2022) 93:930–8. doi: 10.1136/jnnp-2021-328164 PMC9380478 35772923

[33] Dunk MM Li J Liu S Casanova R Chen JC Espeland MA. Associations of dietary cholesterol and fat, blood lipids, and risk for dementia in older women vary by APOE genotype. Alzheimers Dement. (2023) 19:5742–54. doi: 10.1002/alz.13358 PMC10784407 37438877

[34] Musteikytė G Jayaram AK Xu CK Vendruscolo M Krainer G Knowles TPJ. Interactions of α-synuclein oligomers with lipid membranes. Biochim Biophys Acta Biomembr. (2021) 1863:183536. doi: 10.1016/j.bbamem.2020.183536 33373595

[35] Ugalde CL Lawson VA Finkelstein DI Hill AF. The role of lipids in α-synuclein misfolding and neurotoxicity. J Biol Chem. (2019) 294:9016–28. doi: 10.1074/jbc.REV119.007500 PMC6556586 31064841

[36] Dou T Matveyenka M Kurouski D. Elucidation of secondary structure and toxicity of α-synuclein oligomers and fibrils grown in the presence of phosphatidylcholine and phosphatidylserine. ACS Chem Neurosci. (2023) 14:3183–91. doi: 10.1021/acschemneuro.3c00314 PMC10862479 37603792

[37] Qiu J Wei L Su Y Tang Y Peng G Wu Y. Lipid metabolism disorder in cerebrospinal fluid related to Parkinson's disease. Brain Sci. (2023) 13:1166. doi: 10.3390/brainsci13081166 PMC10452343 37626522

[38] Kim WS Halliday GM. Changes in sphingomyelin level affect alpha-synuclein and ABCA5 expression. J Parkinsons Dis. (2012) 2:41–6. doi: 10.3233/JPD-2012-11059 23939407

[39] Jo E McLaurin J Yip CM St George-Hyslop P Fraser PE. alpha-Synuclein membrane interactions and lipid specificity. J Biol Chem. (2000) 275:34328–34. doi: 10.1074/jbc.M004345200 10915790

[40] Canhada S Castro K Perry IS Luft VC. Omega-3 fatty acids' supplementation in Alzheimer's disease: A systematic review. Nutr Neurosci. (2018) 21:529–38. doi: 10.1080/1028415X.2017.1321813 28466678

[41] Bianchi VE Herrera PF Laura R. Effect of nutrition on neurodegenerative diseases. A systematic review. Nutr Neurosci. (2021) 24:810–34. doi: 10.1080/1028415X.2019.1681088 31684843

[42] Fiol-deRoque MA Gutierrez-Lanza R Terés S Torres M Barceló P Rial RV. Cognitive recovery and restoration of cell proliferation in the dentate gyrus in the 5XFAD transgenic mice model of Alzheimer's disease following 2-hydroxy-DHA treatment. Biogerontology. (2013) 14:763–75. doi: 10.1007/s10522-013-9461-4 24114505

