# Supplementary material for: Exploring the causal effects of serum lipids and lipidomes on lewy body dementia: a Mendelian randomization study
Source: Front Endocrinol (Lausanne). 2024 Sep 19;15:1456005. doi: 10.3389/fendo.2024.1456005 (PMC11446761; doi:10.3389/fendo.2024.1456005)

**Figure Legend**

**Figure S1. Results of causal effect and sensitivity analysis of LDL-C on LBD**

**Figure S2. Results of causal effect and sensitivity analysis of RC on LBD**

**Figure S3. Results of causal effect and sensitivity analysis of RC on APOE4 LBD**

**Figure S4. Results of causal effect and sensitivity analysis of PC (O-16:0_20:4) on LBD**

**Figure S5. Results of causal effect and sensitivity analysis of PC (O-18:1_20:4) on LBD**

**Figure S6. Results of causal effect and sensitivity analysis of PI (18:1_20:4) on LBD**

**Figure S7. Results of causal effect and sensitivity analysis of PC (16:1_18:0) on APOE4 LBD**

**Figure S8. Results of causal effect and sensitivity analysis of PC (O-16:1_18:0) on APOE4 LBD**

**Figure S9. Results of causal effect and sensitivity analysis of PC (O-18:2_18:1) on APOE4 LBD**

**Figure S10. Results of causal effect and sensitivity analysis of PE (O-18:2_18:1) on APOE4 LBD**

**Figure S11. Results of causal effect and sensitivity analysis of SM (d38:2) on APOE4 LBD**

**Figure S12. Results of causal effect and sensitivity analysis of TAG (56:5) on APOE4 LBD**

Figure S1

Results of causal effect and sensitivity analysis of LDL-C on LBD
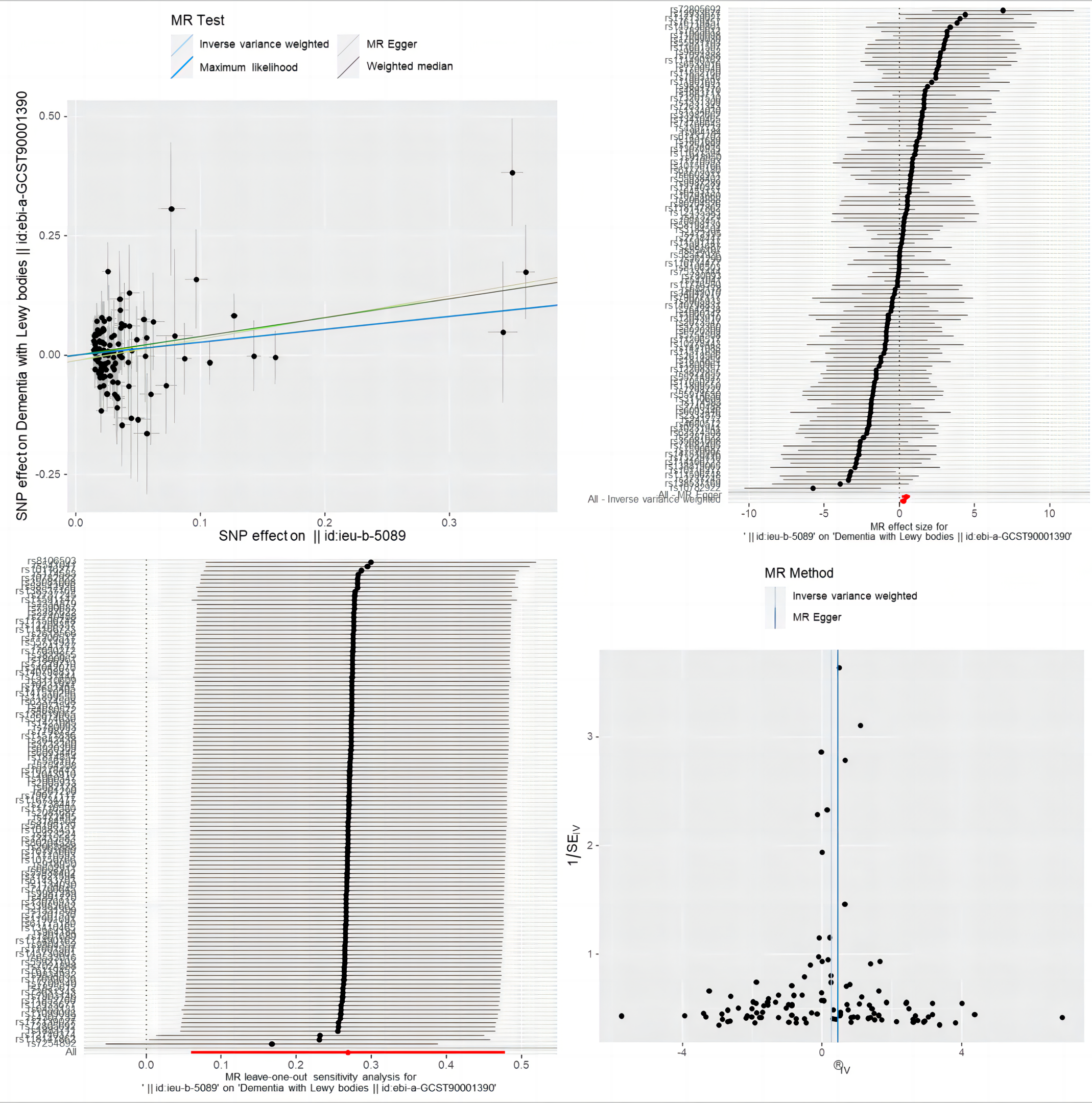


Figure S2

Results of causal effect and sensitivity analysis of RC on LBD


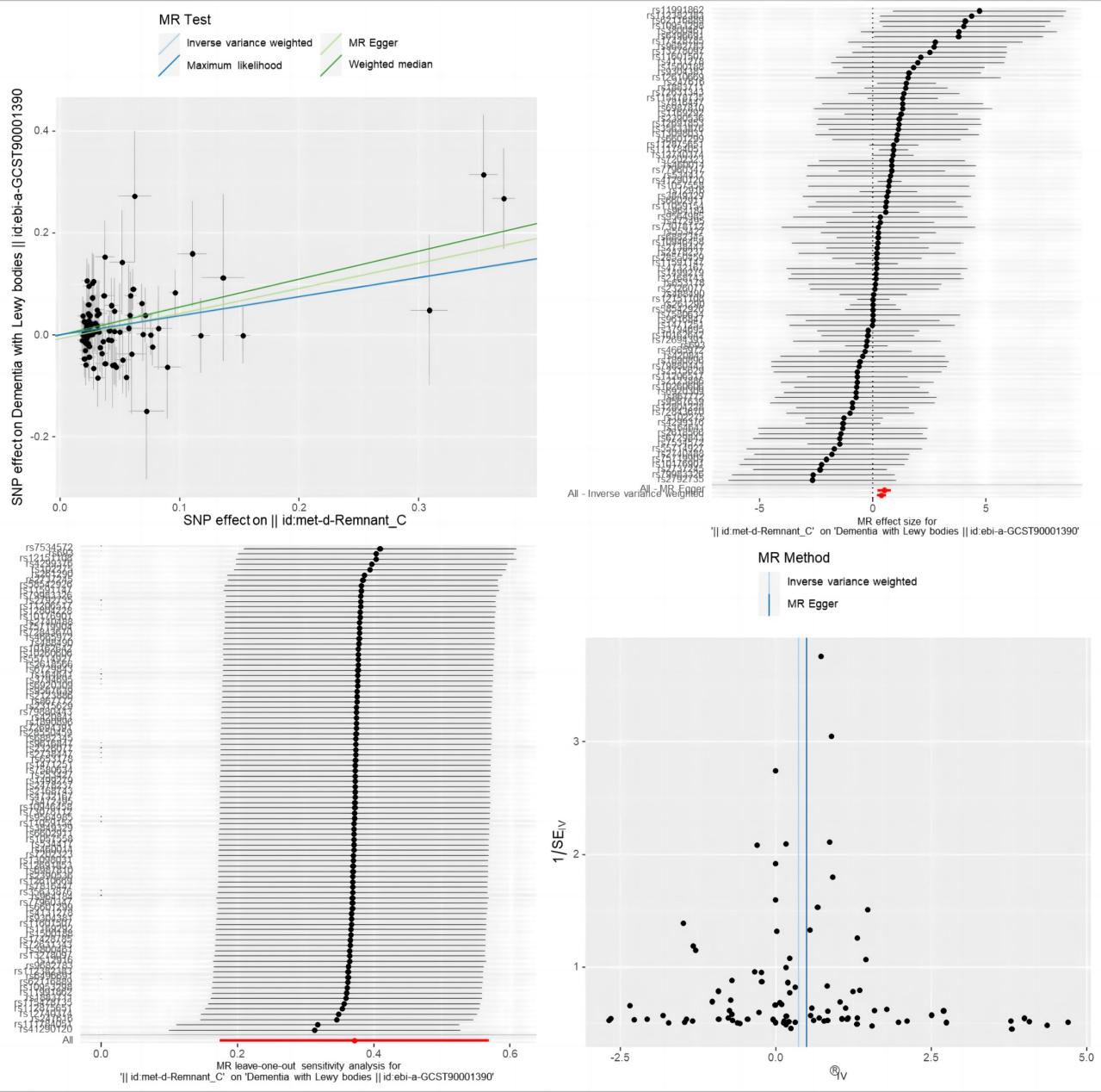


Figure S3

Results of causal effect and sensitivity analysis of RC on APOE4 LBD


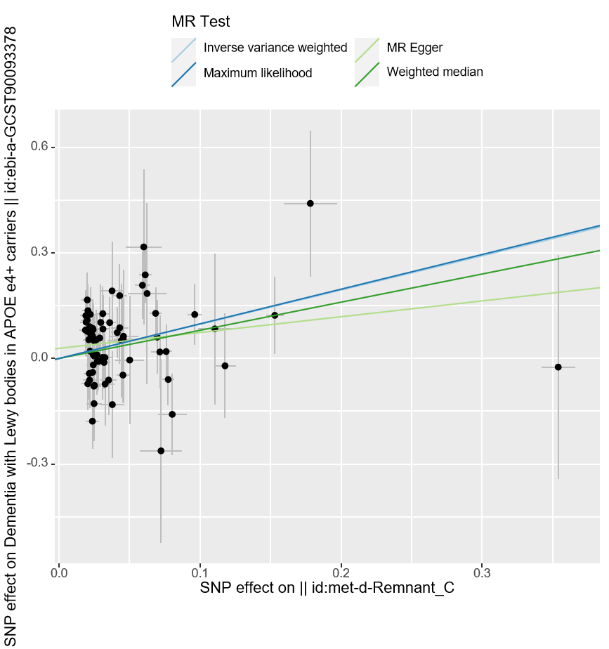

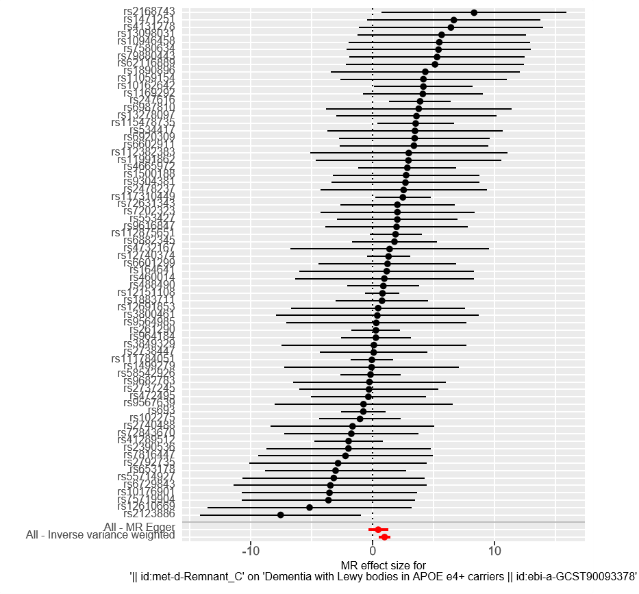


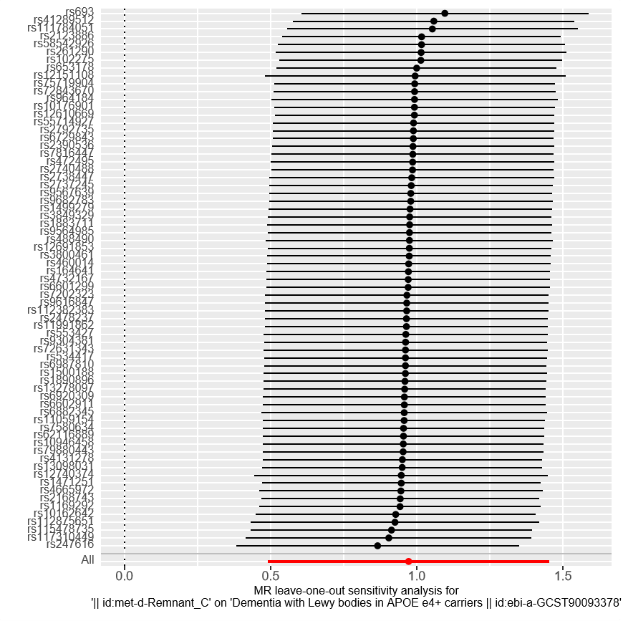

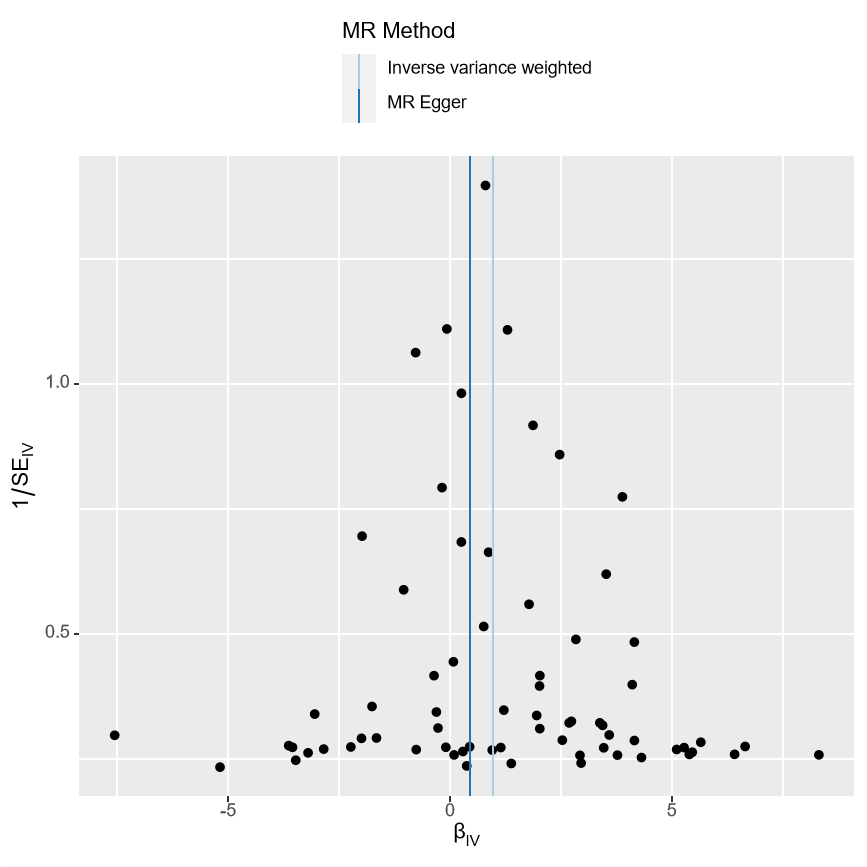


Figure S4

Results of causal effect and sensitivity analysis of PC (O-16:0_20:4) on LBD


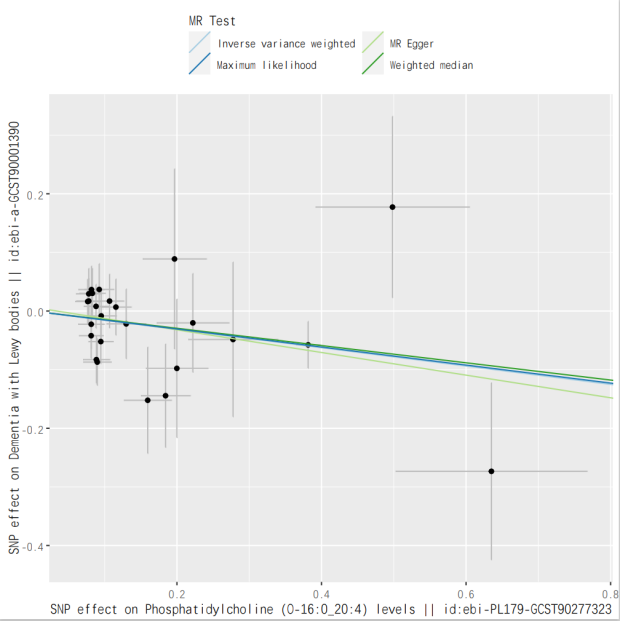

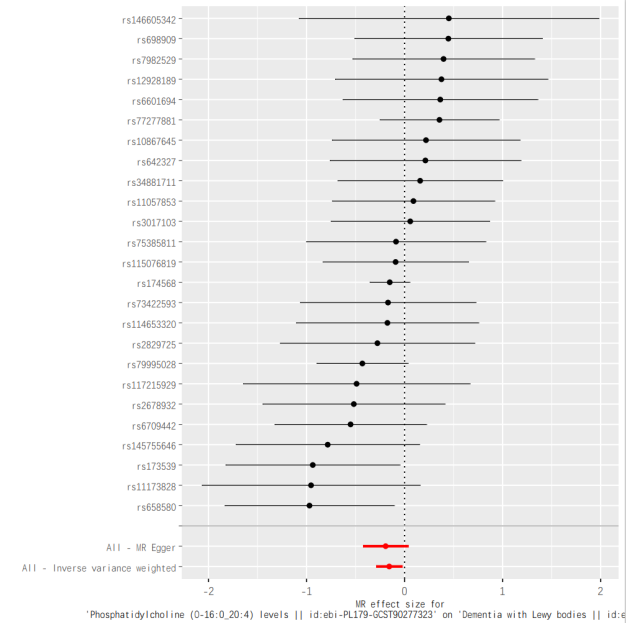


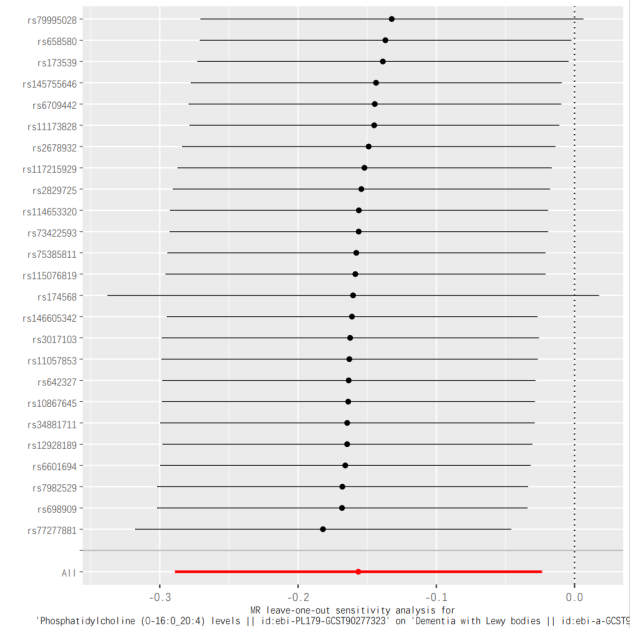

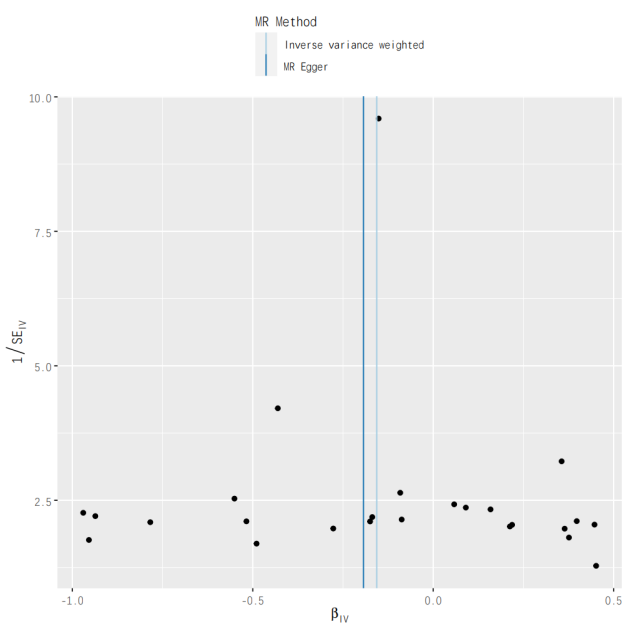


Figure S5

Results of causal effect and sensitivity analysis of PC (O-18:1_20:4) on LBD


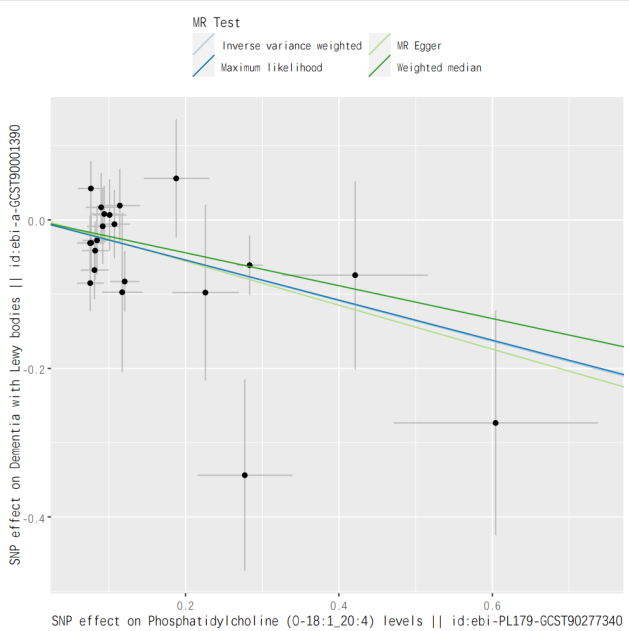

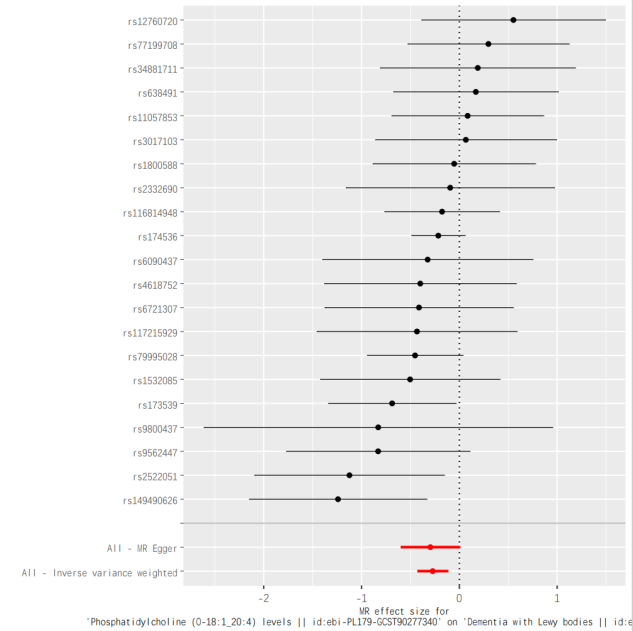


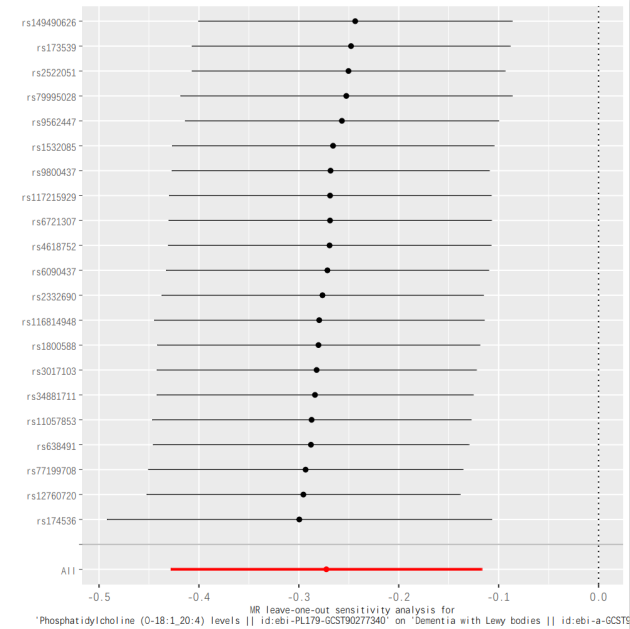

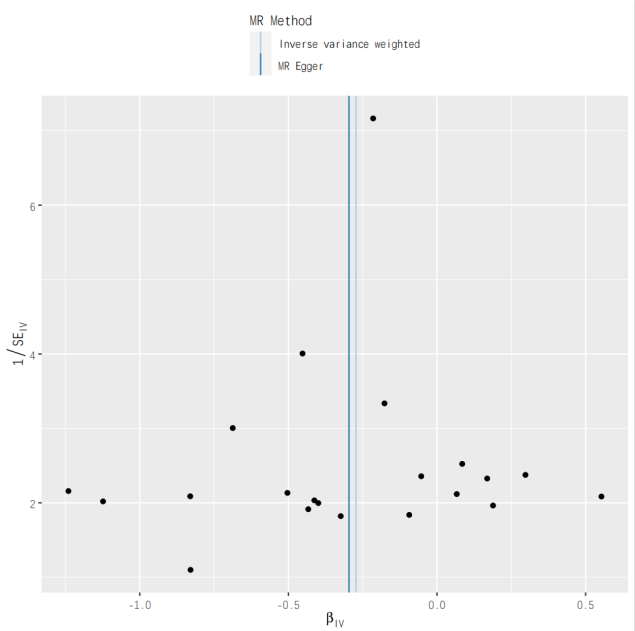


Figure S6

Results of causal effect and sensitivity analysis of PI (18:1_20:4) on LBD


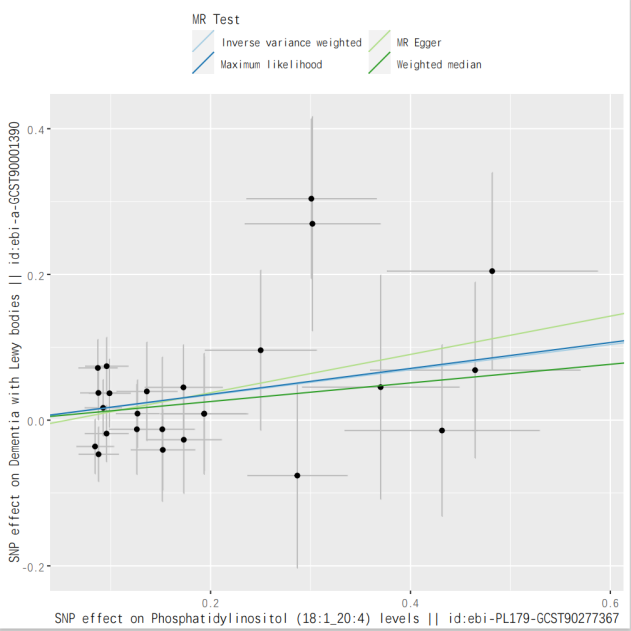

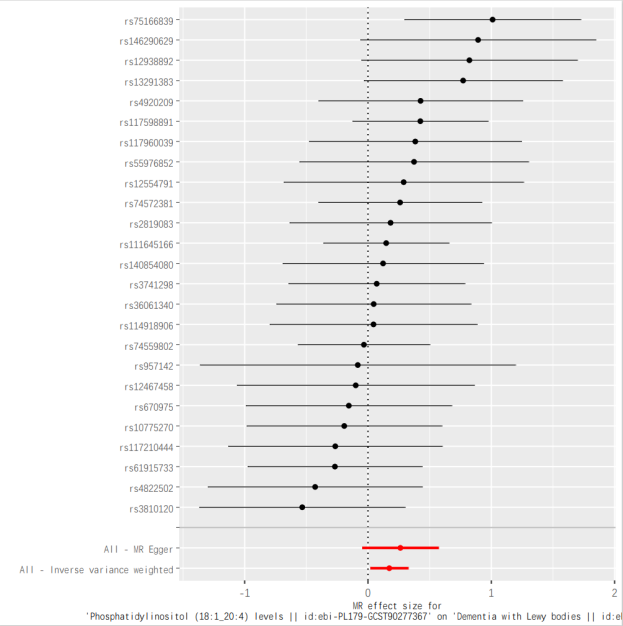


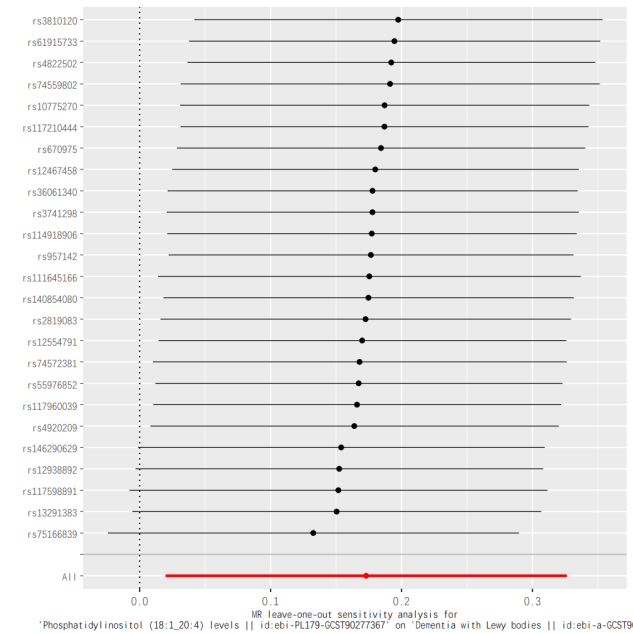

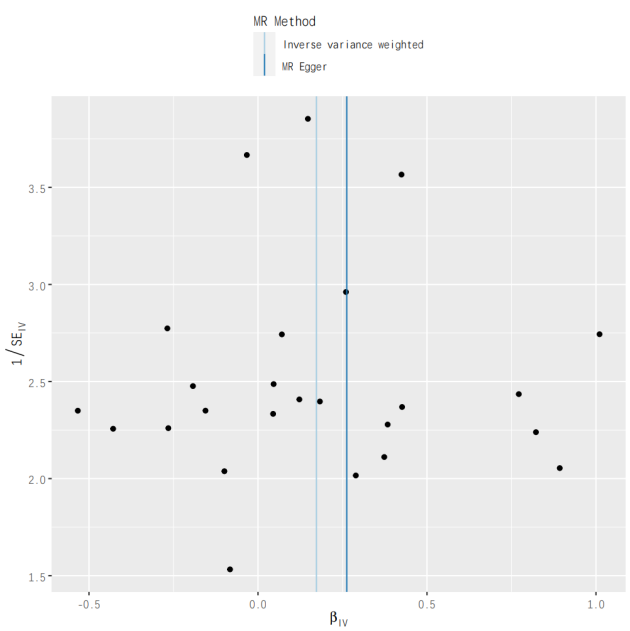


Figure S7

Results of causal effect and sensitivity analysis of PC (16:1_18:0) on APOE4 LBD


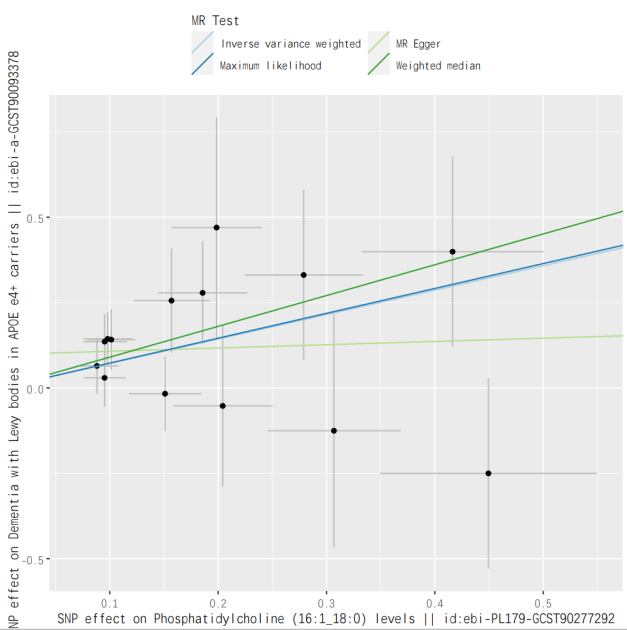

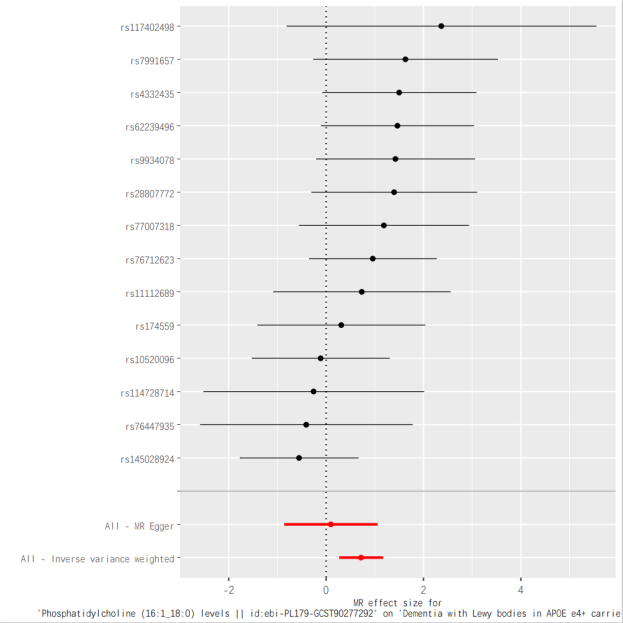


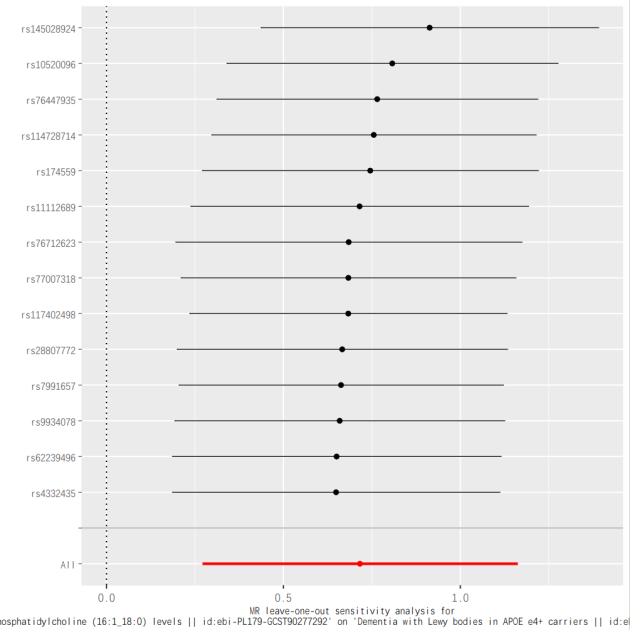

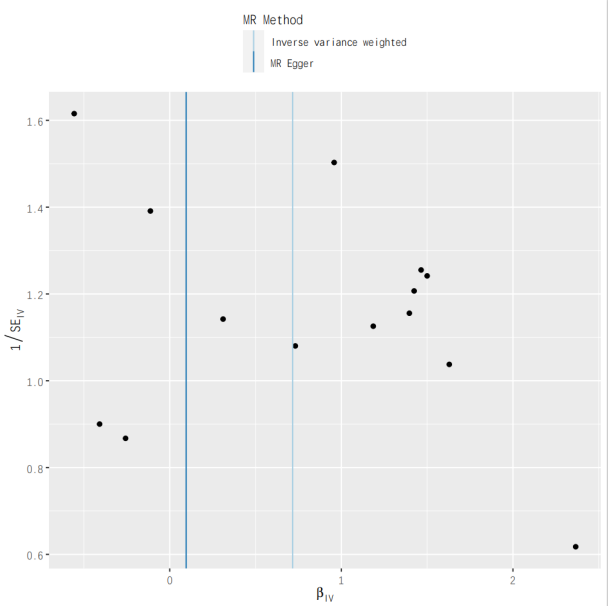


Figure S8

Results of causal effect and sensitivity analysis of PC (O-16:1_18:0) on APOE4 LBD


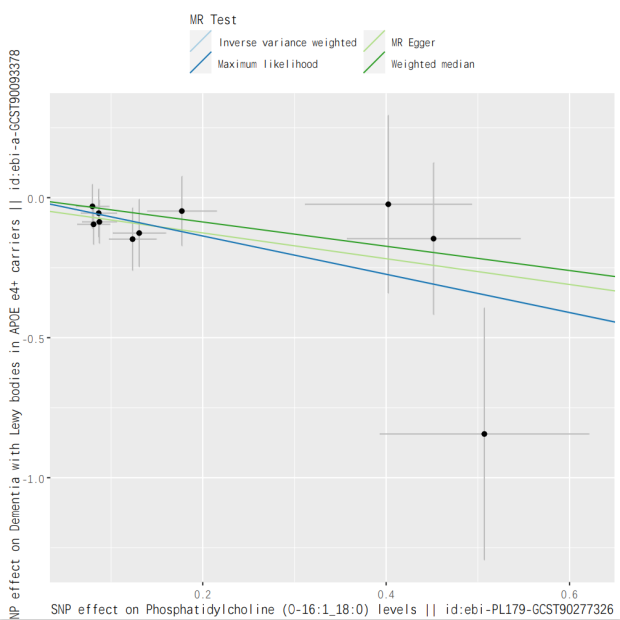

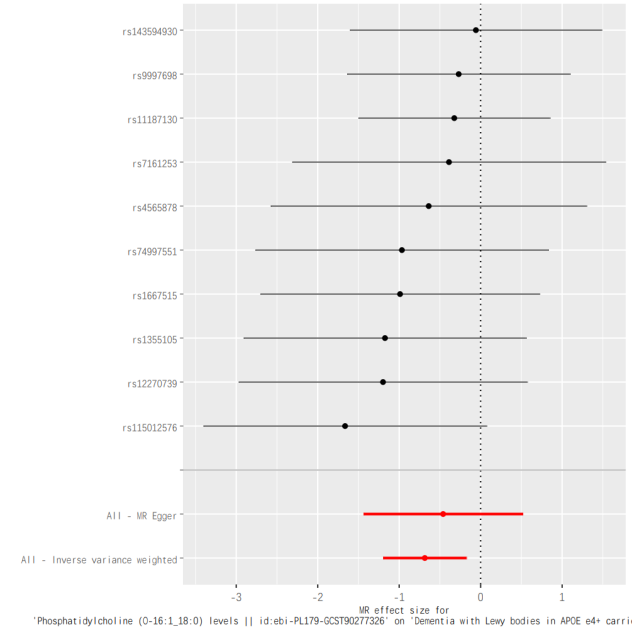


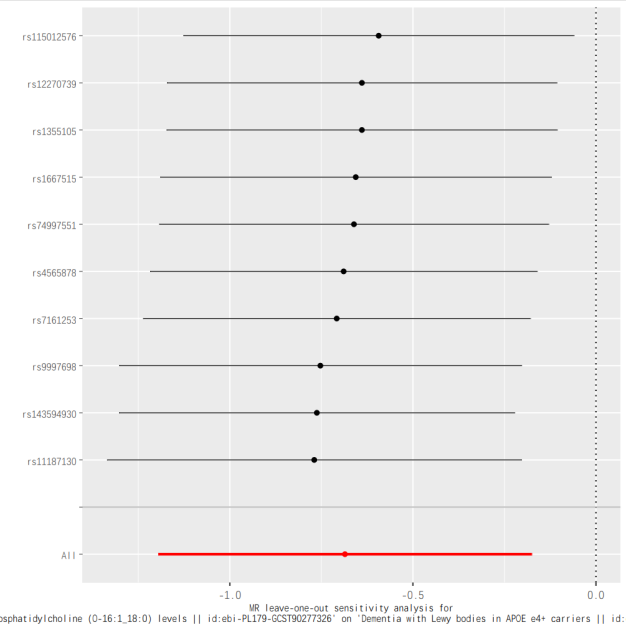

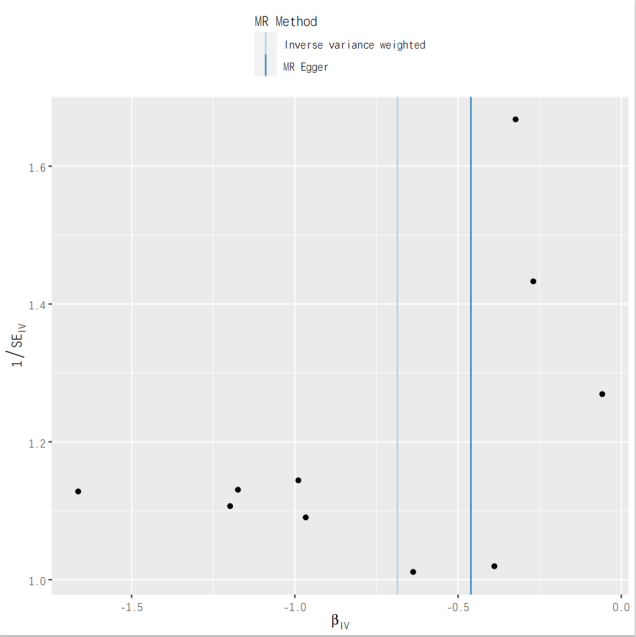


Figure S9

Results of causal effect and sensitivity analysis of PC (O-18:2_18:1) on APOE4 LBD


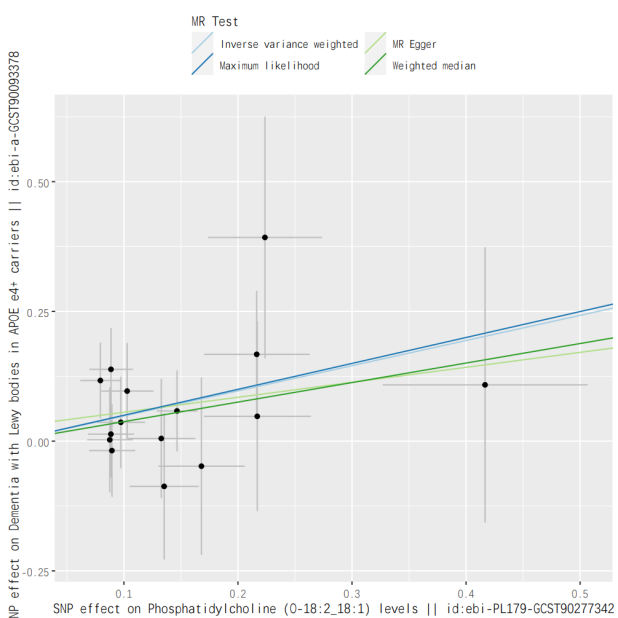

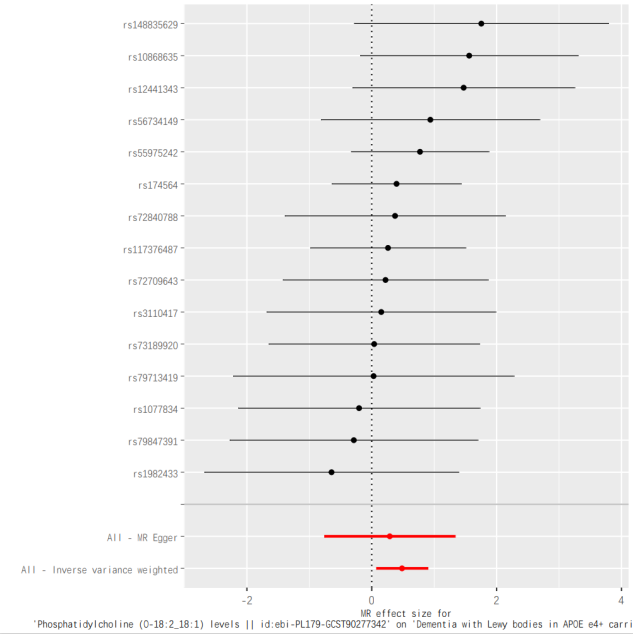


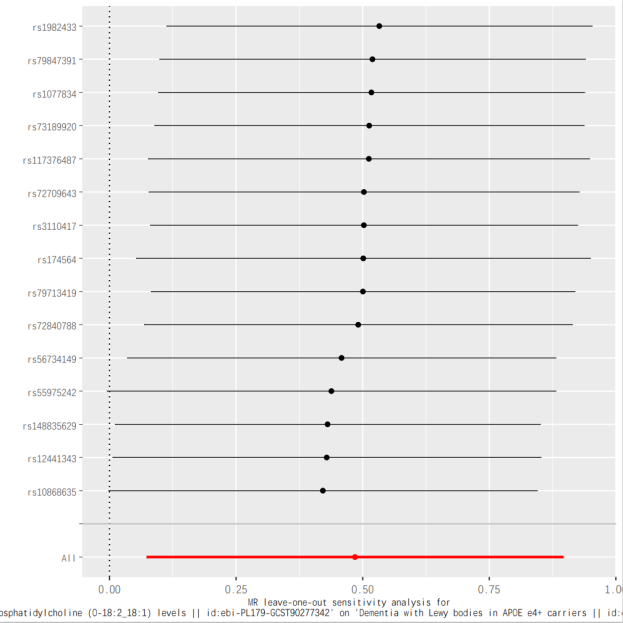

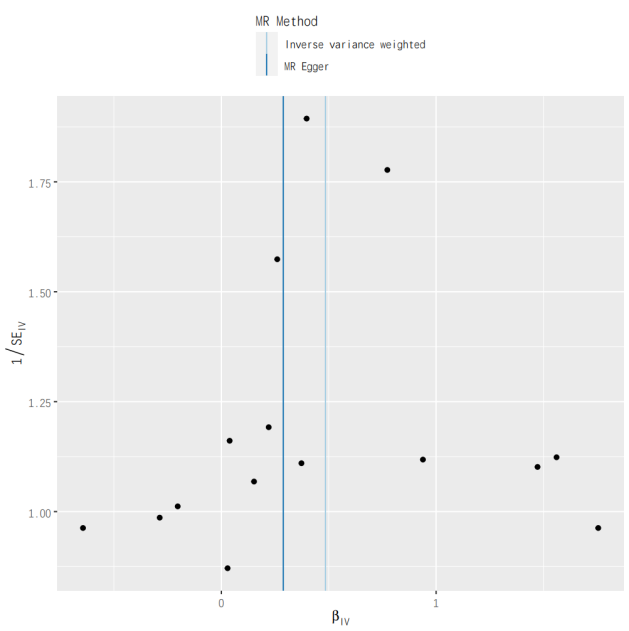


Figure S10

Results of causal effect and sensitivity analysis of PE (O-18:2_18:1) on APOE4 LBD


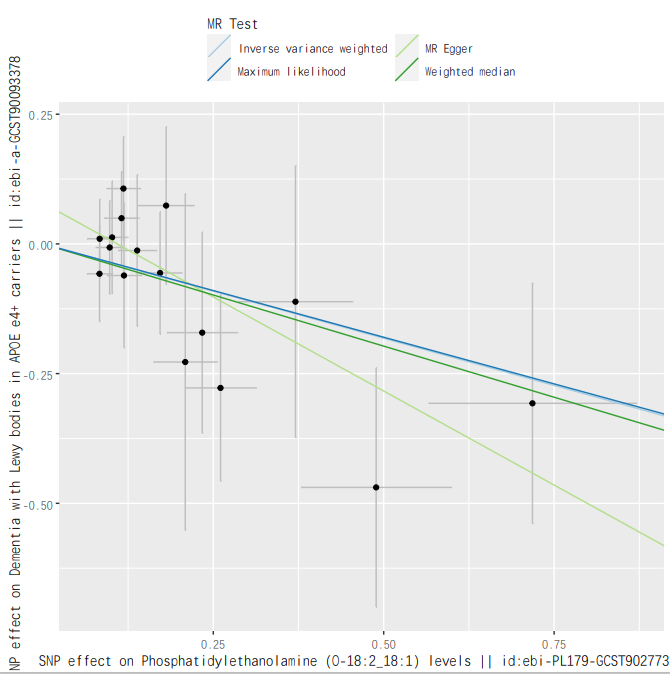

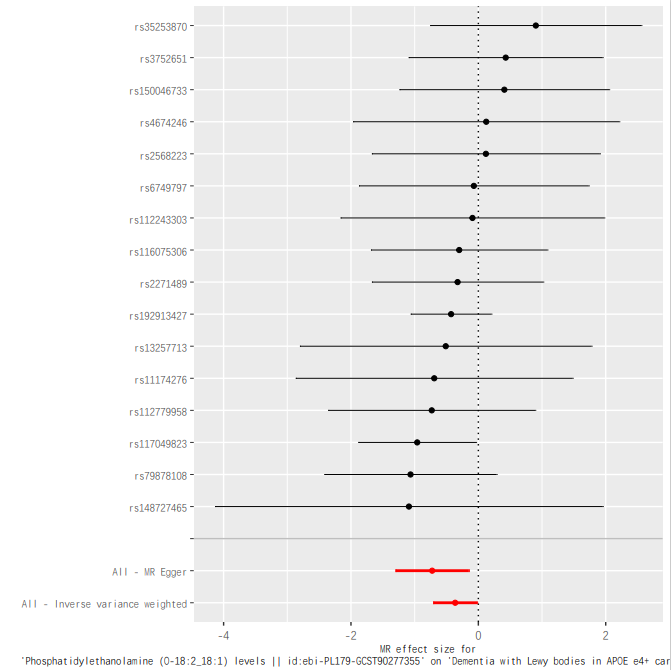


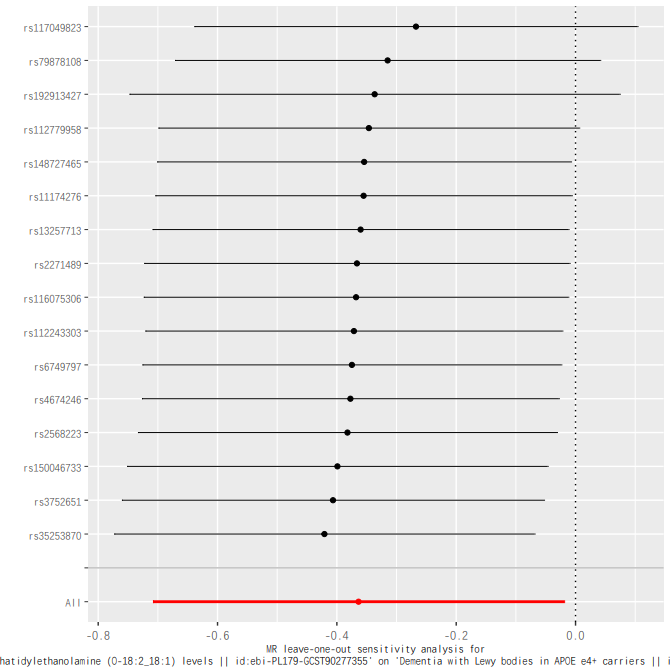

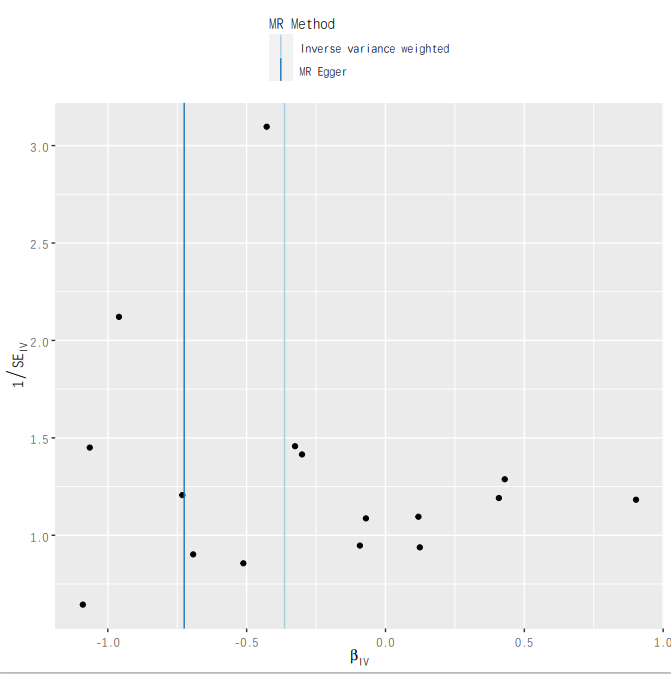


Figure S11

Results of causal effect and sensitivity analysis of SM (d38:2) on APOE4 LBD


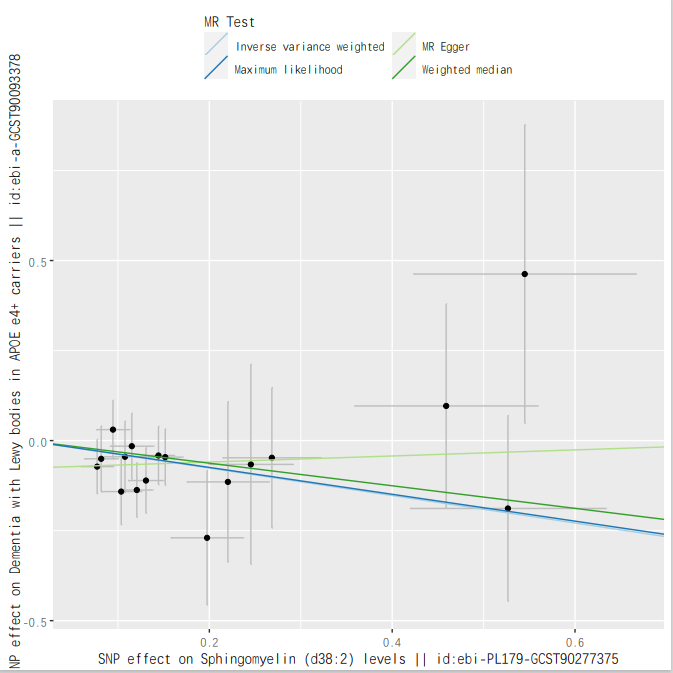

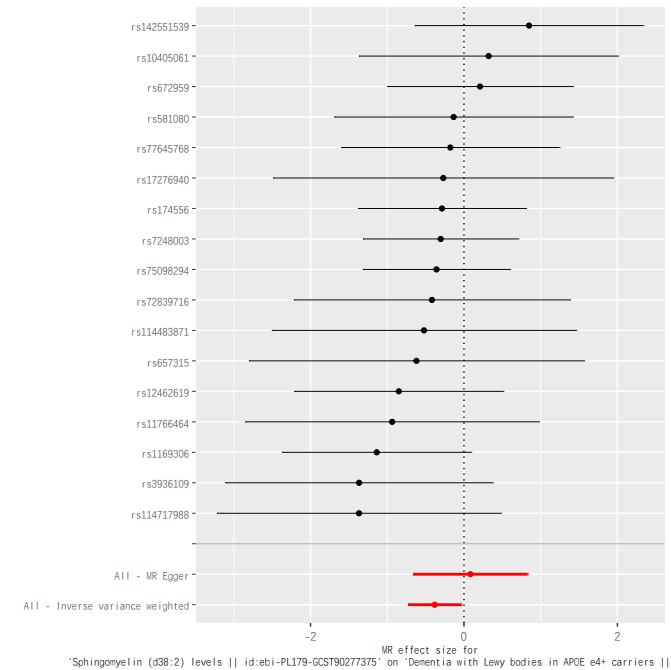


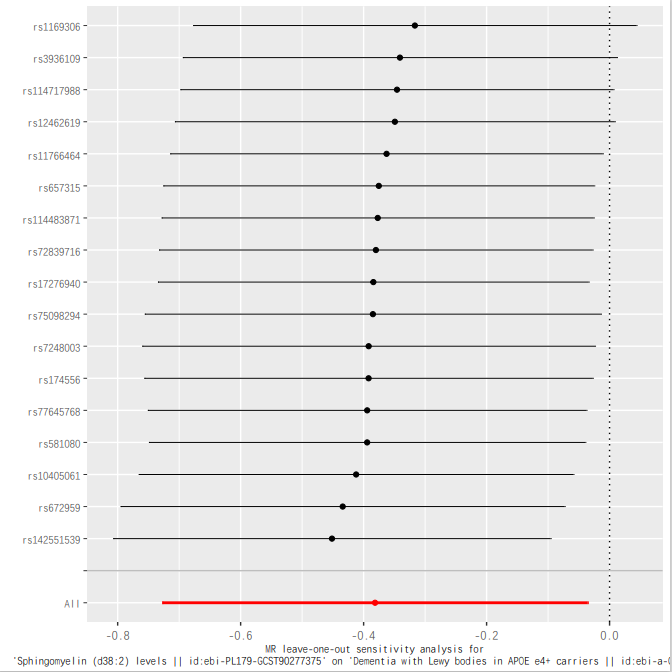

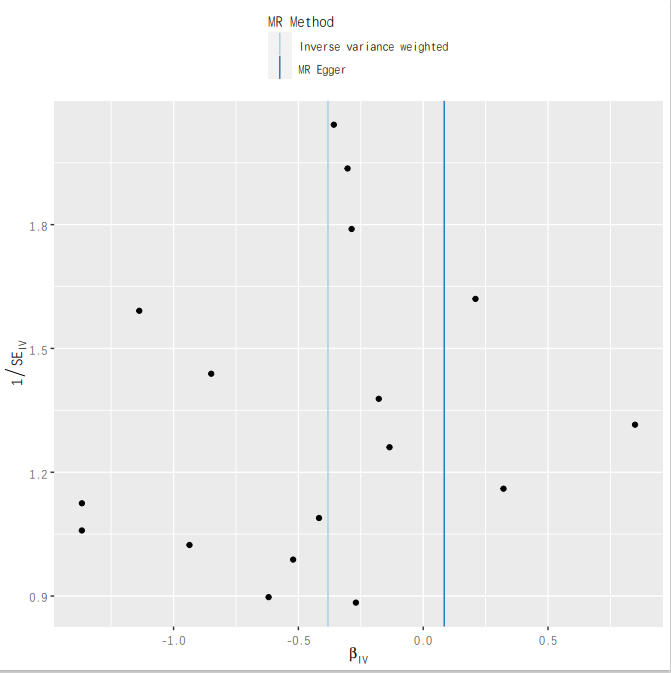


Figure S12

Results of causal effect and sensitivity analysis of TAG (56:5) on APOE4 LBD


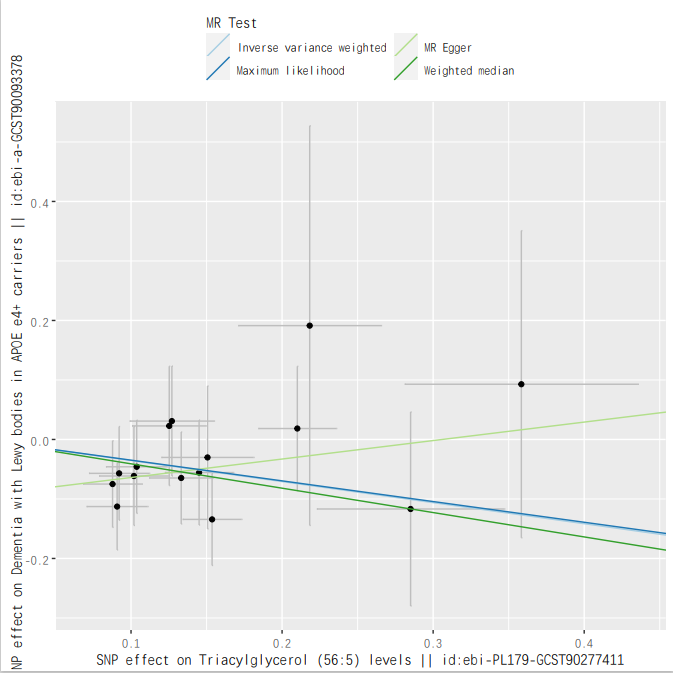

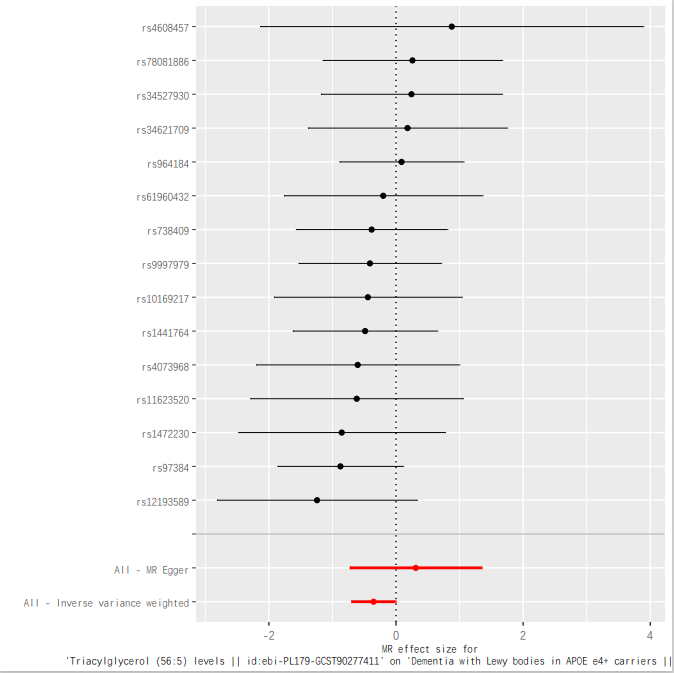


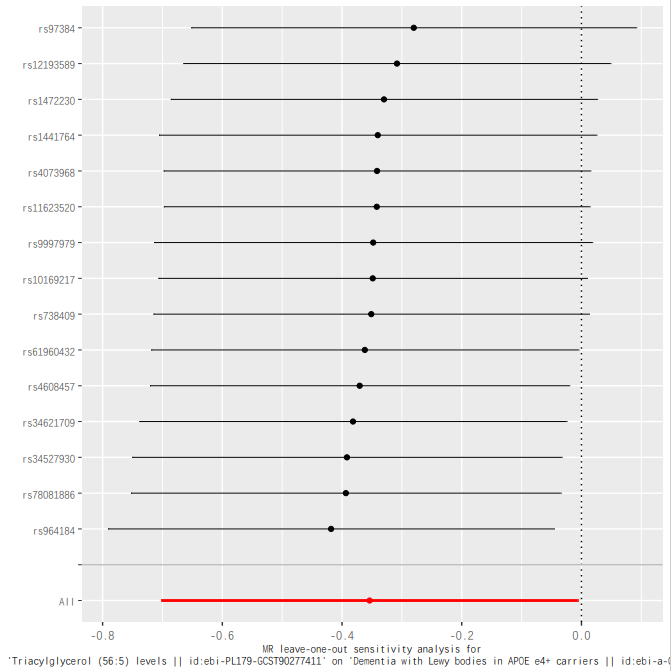

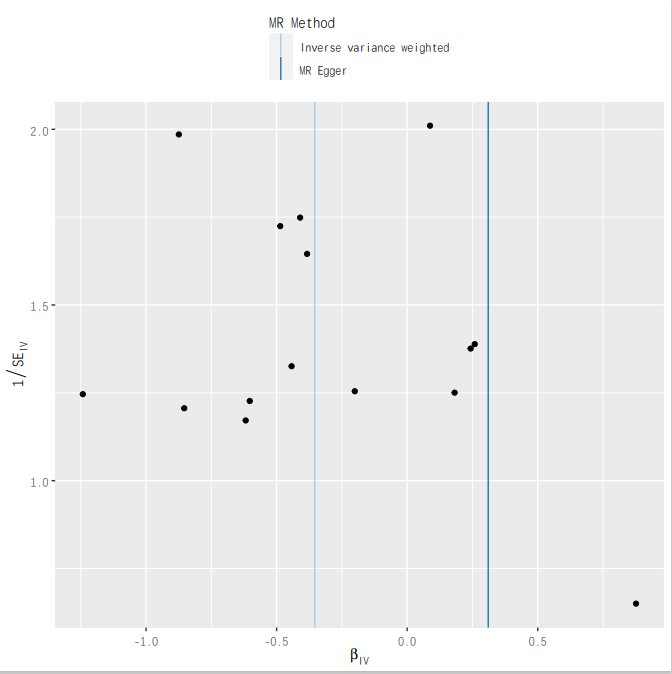

Supplement: Supplementary file 1 [file DataSheet1.docx]
